# Supplementary material for: Diagnostic accuracy of cervical cancer screening and screening–triage strategies among women living with HIV-1 in Burkina Faso and South Africa: A cohort study
Source: PLoS Med. 2021 Mar 4;18(3):e1003528. doi: 10.1371/journal.pmed.1003528 (PMC7971880; doi:10.1371/journal.pmed.1003528)
Supplement: S1 Checklist — (DOCX) [file pmed.1003528.s001.docx]

**STARD (STAndards for the Reporting of Diagnostic accuracy studies) Checklist**

A checklist for reporting of studies of diagnostic accuracy *(version January 2003).* You must report the page number in your manuscript where you consider each of the items listed in this checklist. If you have not included this information, either revise your manuscript accordingly before submitting or note N/A.

| **Section and Topic** | **Item** |  | **paragraph** |
| --- | --- | --- | --- |
| TITLE/ABSTRACT/ KEYWORDS | 1 | [Identify the article as a study of diagnostic accuracy (recommend MeSH heading 'sensitivity and specificity').](http://www.stard-statement.org/item1_maintext.htm)  *This study sought to evaluate the sensitivity and specificity of visual inspection, cervical cytology and HPV-based test for the detection of histology-verified CIN2+/CIN3+ and has the relevant keywords included.* | Title,  Abstract: paragraph 1, Introduction: paragraph 4 |
| INTRODUCTION | 2 | [State the research questions or study aims, such as estimating accuracy between tests or across participant groups.](http://www.stard-statement.org/item2_maintext.htm)  *The primary objective of* *the current study was to evaluate the diagnostic accuracy of three screening approaches (index tests): HR-HPV DNA tests, visual inspection and cervical cytology for the detection of prevalent CIN2+/CIN3+ (reference method) in screening and in triage* | Introduction: paragraph 4 |
| METHODS |  |  |  |
| *Participants* | 3 | [Describe the study population: The inclusion and exclusion criteria, setting and locations where the data were collected.](http://www.stard-statement.org/item3_maintext.htm)  [*The inclusion and exclusion criteria, setting and locations are described in Methods, paragraph 1. The study population characteristics are summarised in Results, paragraph 1.*](http://www.stard-statement.org/item3_maintext.htm) | Methods: paragraph 1 |
|  | 4 | [Describe participant recruitment: Was recruitment based on presenting symptoms, results from previous tests, or the fact that the participants had received the index tests or the reference standard?](http://www.stard-statement.org/item4_maintext.htm)  *Consecutive enrolment of 1238 women living with HIV attending routine HIV care in Johannesburg, South Africa and Ouagadougou, Burkina Faso without previous history of treatment of cervical lesions between December 2011 to October 2012* | Methods: paragraph 1 |
|  | 5 | [Describe participant sampling: Was the study population a consecutive series of participants defined by the selection criteria in items 3 and 4? If not, specify how participants were further selected.](http://www.stard-statement.org/item5_maintext.htm)  *Women were enrolled in the study if they satisfied the inclusion/exclusion criteria given in Methods, paragraph 1*. | Methods: paragraph: 1 |
|  | 6 | [Describe data collection: Was data collection planned before the index test and reference standard were performed (prospective study) or after (retrospective study)?](http://www.stard-statement.org/item6_maintext.htm)  *Sociodemographic, behavioural and clinical data (based on clinical exam) were collected prior to conduct of or sample collection for index test and before reference standard was performed.* | Methods: paragraph 2 |
| *Test methods* | 7 | [Describe the reference standard and its rationale.](http://www.stard-statement.org/item7_maintext.htm)  *The reference standard was histology-verified high-grade cervical intraepithelial neoplasia (CIN2+/CIN3+). histology was classified as ‘negative’ (≤CIN1) or ‘positive’ (CIN2+/CIN3+) based on the highest reading across all findings from the 4-quadrant biopsies and endocervical curettage if collected.* | Methods; paragraph 4 |
|  | 8 | [Describe technical specifications of material and methods involved including how and when measurements were taken, and/or cite references for index tests and reference standard.](http://www.stard-statement.org/item8_maintext.htm)  *Index tests: VIA/VILI described in Methods, paragraph 2; HPV test (Hybrid Capture II) and cervical cytology in Methods, paragraph 3. Reference standard histology in Methods, paragraph 4.* | Methods: paragraphs 2, 3, 4 |
|  | 9 | [Definition of and rationale for the units, cut-offs and/or categories of the results of the index tests and the reference standard.](http://www.stard-statement.org/item9_maintext.htm)  *Reference standard: histology was classified as ‘negative’ (≤CIN1) or ‘positive’ (CIN2+/CIN3+) based on the highest reading across all findings from the 4-quadrant biopsies (Methods, paragraph 4).*  *Index tests: cut-offs for the tests summarised in Methods, paragraph 6 (under sub-title “Statistical analysis”), determined according to IARC guidelines (visual inspection), current local practice (cytology) to achieve optimum sensitivity and specificity for CIN2+ (differed in both countries). For HPV test (Hybrid Capture II), the manufacturer defined cut-off was used, but different cut-off were evaluated in the current study and described in Methods, paragraph 6.* . | Methods, paragraph 6 (Statistical analysis) |
|  | 10 | [Describe the number, training and expertise of the persons executing and reading the index tests and the reference standard.](http://www.stard-statement.org/item10_maintext.htm)  *Index tests: A trained nurse/midwife conducted visual inspection (Methods, paragraph 2), cytologist conducted cervical cytology (Methods, paragraph 3), Hybrid Capture II and CareHPV by trained lab technicians (Methods, paragraph 3). Reference test was conducted by histopathologists locally (Methods, paragraph 4)* | Methods: paragraphs 2, 3, 4 |
|  | 11 | [Describe whether or not the readers of the index tests and reference standard were blind (masked) to the results of the other test and describe any other clinical information available to the readers.](http://www.stard-statement.org/item11_maintext.htm)  *All readers of the index tests (VIA, VILI, Cytology, Hybrid Capture II) were blinded to reference test. The pathologist (reader of reference test) was blinded to the index test results.* | Methods: Paragraph 4 |
| *Statistical methods* | 12 | [Describe methods for calculating or comparing measures of diagnostic accuracy, and the statistical methods used to quantify uncertainty (e.g. 95% confidence intervals).](http://www.stard-statement.org/item12_maintext.htm)  *The study analytical plan (S1 text) summarises the planned analyses at study initiation* | Methods paragraph 6 (Statistical analysis)  S1 text |
|  | 13 | [Describe methods for calculating test reproducibility, if done.](http://www.stard-statement.org/item13_maintext.htm)  *Not applicable* | Not applicable |
| RESULTS |  |  |  |
| *Participants* | 14 | [Report when study was done, including beginning and ending dates of recruitment.](http://www.stard-statement.org/item14_maintext.htm)  *Women were enrolled between December 2011 to October 2012* | Method, paragraph 1 |
|  | 15 | [Report clinical and demographic characteristics of the study population (e.g. age, sex, spectrum of presenting symptoms, co morbidity, current treatments, recruitment centers).](http://www.stard-statement.org/item15_maintext.htm)  *Summarised in Results, paragraph 2* | Results: paragraph 2  Table 1 |
|  | 16 | [Report the number of participants satisfying the criteria for inclusion that did or did not undergo the index tests and/or the reference standard; describe why participants failed to receive either test (a flow diagram is strongly recommended).](http://www.stard-statement.org/item16_maintext.htm) | Figure 1 |
| *Test results* | 17 | [Report time interval from the index tests to the reference standard, and any treatment administered between.](http://www.stard-statement.org/item17_maintext.htm)  *The median time from enrolment when index tests were conducted and colposcopy visit when biopsy was taken, if indicated, for histology verification was 2.9 months (interquartile range [IQR]: 2.1-3.8).* | Results, paragraph 1 |
|  | 18 | [Report distribution of severity of disease (define criteria) in those with the target condition; other diagnoses in participants without the target condition.](http://www.stard-statement.org/item18_maintext.htm)  *Severity of cervical disease (reference standard) varied between grades CIN2+ and CIN3+ and prevalence reported in Table 1, stratified by country.* | Table 1 |
|  | 19 | [Report a cross tabulation of the results of the index tests (including indeterminate and missing results) by the results of the reference standard; for continuous results, the distribution of the test results by the results of the reference standard.](http://www.stard-statement.org/item19_maintext.htm)  Data available in Tables 2 and 3 (country data combined). Country stratified data available in supplementary tables 1-4 inclusive. | Tables 2 and 3 of main manuscript; Supplementary Tables 1-4 |
|  | 20 | [Report any adverse events from performing the index tests or the reference standard.](http://www.stard-statement.org/item20_maintext.htm)  *There were no adverse events from performing the index test. Any adverse events related to taking cervical biopsy (sample required for reference test) summarised in Discussion, paragraph 7.* | Discussion, paragraph 7 |
| *Estimates* | 21 | [Report estimates of diagnostic accuracy and measures of statistical uncertainty (e.g. 95% confidence intervals).](http://www.stard-statement.org/item21_maintext.htm)  *Data available in Tables 2 and 3 (country data combined). Country stratified data available in supplementary tables 1-4 inclusive. Results are also reported in the main manuscript (Results, paragraph 5)* | Tables 2 and 3 of main manuscript; Supplementary Tables 1-4  Results, paragraph 5 |
|  | 22 | [Report how indeterminate results, missing data and outliers of the index tests were handled.](http://www.stard-statement.org/item22_maintext.htm)  *Among 1130 women with valid reference standard results (Results, paragraph 1), women with indeterminate or missing results for cytology (representing 3.3% of all enrolled women), VIA/VILI (<0,1%) and HC-II (0.8%) were excluded from the analysis of diagnostic accuracy of those respective tests. The denominators for each index test are given in Tables 2 and 3.*  *The classification of the index tests was binary (for cytology we used two cut-off to define test positivity: ASCUS+ and HSIL+), there were no outliers.* | Results, paragraph 1, Tables 2 and 3 |
|  | 23 | [Report estimates of variability of diagnostic accuracy between subgroups of participants, readers or centers, if done.](http://www.stard-statement.org/item23_maintext.htm)  *Diagnostic accuracy was stratified by country (Supplementary tables 1-4), age (supplementary table 5 and Results, paragraph 10) and by antiretroviral therapy status (Supplementary tables 6 and 7 and Results, paragraph 11)* | Supplementary tables 1-7,  Results, paragraph 10, 11 |
|  | 24 | [Report estimates of test reproducibility, if done.](http://www.stard-statement.org/item24_maintext.htm)  Not applicable | NA |
| DISCUSSION | 25 | [Discuss the clinical applicability of the study findings.](http://www.stard-statement.org/item25_maintext.htm) | Discussion: Paragraphs 1-7 |
