# Supplementary material for: Diagnostic accuracy of cervical cancer screening and screening–triage strategies among women living with HIV-1 in Burkina Faso and South Africa: A cohort study
Source: PLoS Med. 2021 Mar 4;18(3):e1003528. doi: 10.1371/journal.pmed.1003528 (PMC7971880; doi:10.1371/journal.pmed.1003528)
Supplement: S1 Table — (DOCX) [file pmed.1003528.s002.docx]

**S1 Table.** Diagnostic accuracy of screening strategies for detection of **prevalent CIN3+** among 554 unscreened WLHIV in BF

| **Strategy** | **Tests performed, n** | **Test positive (Colposcopies indicated), n** | **CIN3+ identified, n** | **Colposcopies to detect 1 case of CIN3+, n** | **N colpo per 1000 women screened** | **Sensitivity % (95%CI)** | **Specificity (95%CI)** | **PPV (95%CI)** | **1-NPV (95%CI)** | **Sensitivity relative to standard of care*** | **Specificity relative to standard of care*** |
| --- | --- | --- | --- | --- | --- | --- | --- | --- | --- | --- | --- |
| **Standalone tests** |  |  |  |  |  |  |  |  |  |  |  |
| VIA positive | 553 | 116 (21.0) | 9 | 12.9 | 210 | 69.2 (38.6-90.9) | 80.2 (76.6-83.5) | 7.8 (3.6-14.2) | 0.9 (0.2-2.3) | 0.82 (0.62-1.08) | 1.03 (1.02-1.05) |
| VIA or VILI positive (VIA/VILI)^1^ | 553 | 132 (23.9) | 11 | 12.0 | 239 | 84.6 (54.6-98.1) | 77.6 (73.8-81.0) | 8.3 (4.2-14.4) | 0.5 (0.1-1.7) | 1.00 | 1.00 |
| Cytology ≥ASCUS | 532 | 137 (25.8) | 8 | 17.1 | 258 | 72.7 (39.0-94.0) | 75.2 (71.3-78.9) | 5.8 (2.6-11.2) | 0.8 (0.2-2.2) | 0.89 (0.53-1.49) | 0.96 (0.90-1.02) |
| Cytology ≥HSIL | 532 | 24 (4.5) | 5 | 4.8 | 45 | 45.5 (16.7-76.6) | 96.4 (94.4-97.8) | 20.8 (7.1-42.2) | 1.2 (0.4-2.6) | 0.56 (0.27-1.14) | 1.22 (1.17-1.28) |
| HC-II (RLU ≥1) | 548 | 229 (41.8) | 13 | 17.6 | 418 | 100.0 (75.3-100.0) | 59.6 (55.3-63.8) | 5.7 (3.1-9.5) | 0.0 (0.0-1.1) | 1.18 (0.94-1.49) | 0.77 (0.71-0.83) |
| HC-II (RLU ≥5) | 548 | 194 (35.4) | 13 | 14.9 | 354 | 100.0 (75.3-100.0) | 66.2 (62.0-70.2) | 6.7 (3.6-11.2) | 0.0 (0.0-1.0) | 1.18 (0.94-1.49) | 0.84 (0.78-0.90) |
| HC-II (RLU ≥10) | 548 | 181 (33.0) | 13 | 13.9 | 330 | 100.0 (75.3-100.0) | 68.6 (64.5-72.5) | 7.2 (3.9-12.0) | 0.0 (0.0-1.0) | 1.18 (0.94-1.49) | 0.87 (0.82-0.94) |
| HC-II (RLU ≥20) | 548 | 160 (29.2) | 13 | 12.3 | 292 | 100.0 (75.3-100.0) | 72.5 (68.5-76.3) | 8.1 (4.4-13.5) | 0.0 (0.0-0.9) | 1.18 (0.94-1.49) | 0.92 (0.86-0.99) |
| ***Restricted genotyping*** |  |  |  |  |  |  |  |  |  |  |  |
| HPV16^2^ | 546 | 26 (4.8) | 6 | 4.3 | 48 | 46.2 (19.2-74.9) | 96.2 (94.3-97.7) | 23.1 (9.0-43.6) | 1.3 (0.5-2.8) | 0.55 (0.26-1.12) | 1.24 (1.18-1.30) |
| HPV16/18/45^3^ | 546 | 58 (10.6) | 7 | 8.3 | 106 | 53.8 (25.1-80.8) | 90.4 (87.6-92.8) | 12.1 (5.0-23.3) | 1.2 (0.5-2.7) | 0.64 (0.34-1.20) | 1.16 (1.11-1.23) |
| 8 HR types^4^ | 546 | 129 (23.6) | 13 | 9.9 | 236 | 100.0 (75.3-100.0) | 78.2 (74.5-81.7) | 10.1 (5.5-16.6) | 0.0 (0.0-0.9) | 1.18 (0.94-1.49) | 1.01 (0.95-1.07) |
| HPV16/33/35/58^5^ | 546 | 68 (12.5) | 11 | 6.2 | 125 | 84.6 (54.6-98.1) | 89.3 (86.4-91.8) | 16.2 (8.4-27.1) | 0.4 (0.1-1.5) | 1.00 (0.70-1.43) | 1.15 (1.09-1.21) |
| **Triage of HPV positive women^6^** |  |  |  |  |  |  |  |  |  |  |  |
| VIA only | 160 | 49 (30.6) | 9 | 5.4 | 89 | 69.2 (38.6-90.9) | 72.8 (64.8-79.8) | 18.4 (8.8-32.0) | 3.6 (1.0-9.0) | 0.82 (0.62-1.08) | - |
| VIA or VILI positive (VIA/VILI) | 160 | 55 (34.4) | 11 | 5.5 | 100 | 84.6 (54.6-98.1) | 70.1 (62.0-77.3) | 20.0 (10.4-33.0) | 1.9 (0.2-6.7) | 1.00 | - |
| Cytology ≥ASCUS | 160 | 79 (49.4) | 8 | 9.9 | 144 | 72.7 (39.0-94.0) | 52.3 (44.0-60.6) | 10.1 (4.5-19.0) | 3.7 (0.8-10.4) | 0.89 (0.53-1.49) | - |
| Cytology ≥HSIL | 154 | 18 (11.7) | 5 | 3.6 | 34 | 45.5 (16.7-76.6) | 90.9 (85.0-95.1) | 27.8 (9.7-53.5) | 4.4 (1.6-9.4) | 0.56 (0.27-1.14) | - |
| HPV16/18+ or other HR-HPV+ AND reflex ASCUS+^7^ | 156 | 100 (64.1) | 10 | 10.0 | 187 | 83.3 (51.6-97.9) | 37.5 (29.6-45.9) | 10.0 (4.9-17.6) | 3.6 (0.4-12.3) | 1.00 (0.68-1.48) |  |
| HPV16/18+ or other HR-HPV+ AND reflex VIA^8^ | 159 | 83 (52.2) | 12 | 6.9 | 152 | 92.3 (64.0-99.8) | 51.4 (43.0-59.7) | 14.5 (7.7-23.9) | 1.3 (0.0-7.1) | 1.09 (0.81-1.47) |  |

^1^In Burkina Faso, standard of care is VIA/VILI (VI) and is used as reference in relative sensitivity/specificity estimates; ^2^ positive for HC-II (using RLU ≥20) and HPV16 by INNO-LiPA; ^3^ positive for HC-II (using RLU ≥20) and any of HPV16, HPV18 or HPV45 by INNO-LiPA; ^4^ positive for HC-II (using RLU ≥20) and any HPV16/18/45/31/33/35/52/58; ^5^ positive for HC-II (using RLU ≥20) and any HPV16/33/35/58; ^6^calculated among women testing positive for HPV DNA, using HC-II ≥20RLU to define test positive (as sensitivity is equivalent irrespective of threshold used, a higher threshold was used to maximise specificity); ^7^test positive if HPV16 or HPV18 positive, or cytology [ASCUS+] when negative for both HPV16 and HPV18; ^8^test positive if HPV16 or HPV18 positive, or VIA abnormal when negative for both HPV16 and HPV18
