# Supplementary material for: Diagnostic accuracy of cervical cancer screening and screening–triage strategies among women living with HIV-1 in Burkina Faso and South Africa: A cohort study
Source: PLoS Med. 2021 Mar 4;18(3):e1003528. doi: 10.1371/journal.pmed.1003528 (PMC7971880; doi:10.1371/journal.pmed.1003528)
Supplement: S2 Table — (DOCX) [file pmed.1003528.s003.docx]

**S2 Table.** Diagnostic accuracy of screening strategies for detection of **prevalent CIN3+** among 576 unscreened WLHIV in SA

| **Strategy** | **Tests performed, n** | **Test positive (Colposcopies indicated), n** | **CIN3+ identified, n** | **Colposcopies to detect 1 case of CIN3+, n** | **N colpo per 1000 women screened** | **Sensitivity % (95%CI)** | **Specificity (95%CI)** | **PPV (95%CI)** | **1-NPV (95%CI)** | **Sensitivity relative to standard of care*** | **Specificity relative to standard of care*** |
| --- | --- | --- | --- | --- | --- | --- | --- | --- | --- | --- | --- |
| **Standalone tests** |  |  |  |  |  |  |  |  |  |  |  |
| VIA positive | 576 | 162 (28.1) | 28 | 5.8 | 281 | 52.8 (38.6-66.7) | 74.4 (70.4-78.1) | 17.3 (11.8-24.0) | 6.0 (3.9-8.8) | 0.67 (0.52-0.86) | 0.99 (0.93-1.06) |
| VIA or VILI positive (VIA/VILI) | 576 | 239 (41.5) | 35 | 6.8 | 415 | 66.0 (51.7-78.5) | 61.0 (56.7-65.2) | 14.6 (10.4-19.8) | 5.3 (3.2-8.3) | 0.81 (0.65-1.01) | 0.81 (0.75-0.88) |
| Cytology ≥LSIL | 561 | 504 (89.8) | 50 | 10.1 | 898 | 96.2 (86.8-99.5) | 10.8 (8.2-13.8) | 9.9 (7.5-12.9) | 3.5 (0.4-12.1) | 1.19 (1.05-1.34) | 0.14 (0.11-0.18) |
| Cytology ≥HSIL^1^ | 561 | 169 (30.1) | 42 | 4.0 | 301 | 80.8 (67.5-90.4) | 75.0 (71.1-78.8) | 24.9 (18.5-32.1) | 2.6 (1.2-4.6) | 1.00 | 1.00 |
| HC-II (RLU ≥1) | 573 | 342 (59.7) | 44 | 7.8 | 597 | 83.0 (70.2-91.9) | 42.7 (38.4-47.1) | 12.9 (9.5-16.9) | 3.9 (1.8-7.3) | 1.02 (0.89-1.18) | 0.57 (0.52-0.63) |
| HC-II (RLU ≥5) | 573 | 287 (50.1) | 42 | 6.8 | 501 | 79.2 (65.9-89.2) | 52.9 (48.5-57.2) | 14.6 (10.8-19.3) | 3.8 (1.9-6.8) | 1.00 (0.86-1.16) | 0.70 (0.65-0.76) |
| HC-II (RLU ≥10) | 573 | 264(46.1) | 39 | 6.8 | 461 | 73.6 (59.7-84.7) | 56.7 (52.3-61.0) | 14.8 (10.7-19.6) | 4.5 (2.5-7.5) | 0.93 (0.78-1.11) | 0.75 (0.70-0.81) |
| HC-II (RLU ≥20) | 573 | 238 (41.5) | 37 | 6.4 | 415 | 69.8 (55.7-81.7) | 61.3 (57.0-65.6) | 15.5 (11.2-20.8) | 4.8 (2.8-7.6) | 0.88 (0.74-1.05) | 0.81 (0.76-0.87) |
| ***Restricted genotyping*** |  |  |  |  |  |  |  |  |  |  |  |
| HPV16^2^ | 573 | 73 (12.7) | 18 | 4.1 | 127 | 34.0 (21.5-48.3) | 89.4 (86.5-91.9) | 24.7 (15.3-36.1) | 7.0 (4.9-9.6) | 0.43 (0.29-0.63) | 1.19 (1.12-1.26) |
| HPV16/18/45^3^ | 573 | 142 (24.8) | 21 | 6.8 | 247 | 39.6 (26.5-54.0) | 76.7 (72.9-80.3) | 14.8 (9.4-21.7) | 7.4 (5.1-10.3) | 0.50 (0.35-0.70) | 1.03 (0.96-1.09) |
| 8 HR types^4^ | 573 | 297 (51.8) | 43 | 6.9 | 517 | 81.1 (68.0-90.6) | 51.2 (46.8-55.5) | 14.5 (10.7-19.0) | 3.6 (1.8-6.6) | 1.00 (0.86-1.16) | 0.68 (0.63-0.74) |
| HPV16/33/35/58^5^ | 573 | 189 (33.0) | 35 | 5.4 | 329 | 66.0 (51.7-78.5) | 70.4 (66.3-74.3) | 18.5 (13.3-24.8) | 4.7 (2.8-7.3) | 0.81 (0.66-1.00) | 0.93 (0.88-0.99) |
| **Triage of HPV positive women^6^** |  |  |  |  |  |  |  |  |  |  |  |
| VIA only | 342 | 110 (32.2) | 23 | 4.3 | 192 | 52.3 (36.7-67.5) | 70.8 (65.3-75.9) | 20.9 (13.7-29.7) | 9.1 (5.7-13.5) | 0.61 (0.46-0.80) | - |
| VIA or VILI positive (VIA/VILI) | 342 | 163 (47.7) | 29 | 4.9 | 284 | 65.9 (50.1-79.5) | 55.0 (49.2-60.8) | 17.8 (12.3-24.5) | 8.4 (4.8-13.4) | 0.74 (0.59-0.92) | - |
| Cytology ≥LSIL | 333 | 308 (92.5) | 42 | 6.1 | 538 | 97.7 (87.7-99.9) | 8.3 (5.4-12.1) | 13.6 (10.0-18.0) | 4.0 (0.1-20.4) | 1.11 (1.00-1.22) | - |
| Cytology ≥HSIL | 333 | 150 (45.0) | 38 | 4.0 | 363 | 88.4 (74.9-96.1) | 61.4 (55.5-67.0) | 25.3 (18.6-33.1) | 2.7 (0.9-6.3) | 1.00 | - |
| HPV16/18+ or other HR-HPV+ AND reflex HSIL+^7^ | 335 | 208 (62.1) | 41 | 5.1 | 370 | 95.3 (84.2-99.4) | 42.8 (37.1-48.7) | 19.7 (14.5-25.8) | 1.6 (0.2-5.6) | 1.08 (0.99-1.18) |  |
| HPV16/18+ or other HR-HPV+ AND reflex VIA ^8^ | 342 | 180 (52.6) | 32 | 5.6 | 314 | 72.7 (57.2-85.0) | 50.3 (44.5-56.2) | 17.8 (12.5-24.2) | 7.4 (3.9-12.6) | 0.84 (0.68-1.04) |  |

^1^In South Africa, standard of care used is cytology; HSIL+ is used as reference in relative sensitivity/specificity estimates; ^2^ positive for HC-II (using RLU ≥1) and HPV16 by INNO-LiPA; ^3^ positive for HC-II (using RLU ≥1) and any of HPV16, HPV18 or HPV45 by INNO-LiPA; ^4^ positive for HC-II (using RLU ≥1) and any HPV16/18/45/31/33/35/52/58; ^5^ positive for HC-II (using RLU ≥1) and any HPV16/33/35/58; ^6^calculated among women testing positive for HPV DNA, using HC-II ≥1RLU to define test positive (maximum sensitivity achieved using ≥1RLU to define test positivity in SA); ^7^test positive if HPV16 or HPV18 positive, or cytology [HSIL+] when negative for both HPV16 and HPV18; ^8^test positive if HPV16 or HPV18 positive, or VIA abnormal when negative for both HPV16 and HPV18
