# Supplementary material for: Diagnostic accuracy of cervical cancer screening and screening–triage strategies among women living with HIV-1 in Burkina Faso and South Africa: A cohort study
Source: PLoS Med. 2021 Mar 4;18(3):e1003528. doi: 10.1371/journal.pmed.1003528 (PMC7971880; doi:10.1371/journal.pmed.1003528)
Supplement: S3 Table — (DOCX) [file pmed.1003528.s004.docx]

**S3 Table.** Diagnostic accuracy of screening strategies for detection of **prevalent CIN2+** among 554 unscreened WLHIV in BF

| **Strategy** | **Tests performed, n** | **Test positive (Colposcopies indicated),**  **n (%)** | **CIN2+ identified, n** | **Colposcopies to detect 1 case of CIN2+, n** | **N colpo per 1000 women screened** | **Sensitivity % (95%CI)** | **Specificity (95%CI)** | **PPV (95%CI)** | **1-NPV (95%CI)** | **Sensitivity relative to standard of care^1^** | **Specificity relative to standard of care^1^** |
| --- | --- | --- | --- | --- | --- | --- | --- | --- | --- | --- | --- |
| **Standalone tests** |  |  |  |  |  |  |  |  |  |  |  |
| VIA positive only | 553 | 116 (21.0) | 14 | 8.3 | 210 | 43.8 (26.4-62.3) | 80.4 (76.8-83.7) | 12.1 (6.8 (19.4) | 4.1 (2.5-6.4) | 0.78 (0.61-1.00) | 1.03 (1.01-1.05) |
| VIA or VILI positive (VIA/VILI)^1^ | 553 | 132 (23.9) | 18 | 7.3 | 239 | 56.3 (37.3-73.6) | 78.1 (74.3-81.6) | 13.6 (8.3-20.7) | 3.3 (1.8-5.5) | 1.00 | 1.00 |
| Cytology ≥ASCUS | 532 | 137 (25.8) |  | 6.0 | 258 | 76.7 (57.7-90.1) | 77.3 (73.4-80.9) | 16.8 (11.0-24.1) | 1.8 (0.7-3.6) | 1.44 (0.99-2.08) | 0.97 (0.92-1.03) |
| Cytology ≥HSIL | 532 | 24 (4.5) | 9 | 2.7 | 45 | 30.0 (14.7-49.4) | 97.0 (95.1-98.3) | 37.5 (18.8-59.4) | 4.1 (2.6-6.2) | 0.56 (0.33-0.97) | 1.22 (1.17-1.28) |
| HC-II (RLU ≥1) | 548 | 229 (41.8) | 31 | 7.4 | 418 | 96.9 (83.8-99.9) | 61.6 (57.3-65.8) | 13.5 (9.4-18.7) | 0.3 (0.0-1.7) | 1.72 (1.28-2.32) | 0.79 (0.73-0.85) |
| HC-II (RLU ≥5) | 548 | 194 (35.4) | 31 | 6.3 | 354 | 96.9 (83.8-99.9) | 68.4 (64.2-72.4) | 16.0 (11.1-21.9) | 0.3 (0.0-1.6) | 1.72 (1.28-2.32) | 0.86 (0.81-0.93) |
| HC-II (RLU ≥10) | 548 | 181 (33.0) | 31 | 5.8 | 330 | 96.9 (83.8-99.9) | 70.9 (66.8-74.8) | 17.1 (11.9-23.4) | 0.3 (0.0-1.5) | 1.72 (1.28-2.32) | 0.90 (0.84-0.96) |
| HC-II (RLU ≥20) | 548 | 160 (29.2) | 31 | 5.2 | 292 | 96.9 (83.8-99.9) | 75.0 (71.0-78.7) | 19.4 (13.6-26.4) | 0.3 (0.0-1.4) | 1.72 (1.28-2.32) | 0.95 (0.89-1.01) |
| ***Restricted genotyping*** |  |  |  |  |  |  |  |  |  |  |  |
| HPV16^2^ | 546 | 26 (4.8) | 11 | 2.4 | 48 | 35.5 (19.2-54.6) | 97.1 (95.2-98.4) | 42.3 (23.4-63.1) | 3.8 (2.4-5.9) | 0.61 (0.33-1.12) | 1.24 (1.18-1.30) |
| HPV16/18/45^3^ | 546 | 58 (10.6) | 15 | 3.9 | 106 | 48.4 (30.2-66.9) | 91.7 (88.9-93.9) | 25.9 (15.3-39.0) | 3.3 (1.9-5.3) | 0.83 (0.50-1.40) | 1.17 (1.11-1.23) |
| 8 HR types^4^ | 546 | 129 (23.6) | 29 | 4.4 | 236 | 93.5 (78.6-99.2) | 80.6 (76.9-83.9) | 22.5 (15.6-30.7) | 0.5 (0.1-1.7) | 1.61 (1.21-2.14) | 1.03 (0.97-1.09) |
| HPV16/33/35/58^5^ | 546 | 68 (12.5) | 23 | 3.0 | 125 | 74.2 (55.4-88.1) | 91.3 (88.5-93.6) | 33.8 (22.8-46.3) | 1.7 (0.7-3.3) | 1.28 (0.88-1.86) | 1.17 (1.11-1.23) |
| **Triage of HPV positive women^6^** |  |  |  |  |  |  |  |  |  |  |  |
| VIA positive only | 160 | 49 (30.6) | 14 | 3.5 | 89 | 43.2 (27.3-64.0) | 72.9 (64.3-80.3) | 28.6 (16.6-43.3) | 15.3 (9.2-23.4) | 0.78 (0.61-1.00) | - |
| VIA or VILI positive (VIA/VILI) | 160 | 55 (34.4) | 18 | 3.1 | 100 | 58.1 (39.1-75.5) | 71.3 (62.7-78.9) | 32.7 (20.7-46.7) | 12.4 (6.8-20.2) | 1.00 | - |
| Cytology ≥ASCUS | 160 | 79 (49.4) | 22 | 3.6 | 144 | 75.9 (56.5-89.7) | 56.5 (47.6-65.1) | 27.8 (18.3-39.1) | 8.6 (3.5-17.0) | 1.38 (0.96-1.97) | - |
| Cytology ≥HSIL | 154 | 18 (11.7) | 9 | 2.0 | 34 | 31.0 (15.3-50.8) | 92.8 (86.8-96.7) | 50.0 (26.0-74.0) | 14.7 (9.2-21.8) | 0.56 (0.33-0.97) | - |
| HPV16/18+ or other HR-HPV+ AND reflex ASCUS+^7^ | 156 | 100 (64.1) | 27 | 3.7 | 187 | 90.0 (73.5-97.9) | 42.1 (33.3-51.2) | 27.0 (18.6-36.8) | 5.4 (1.1-14.9) | 1.59 (1.13-2.24) |  |
| HPV16/18+ or other HR-HPV+ AND reflex VIA^8^ | 159 | 83 (52.2) | 23 | 3.6 | 152 | 76.7 (57.7-90.1) | 53.5 (44.5-62.3) | 27.7 (18.4-38.6) | 9.2 (3.8-18.1) | 1.28 (0.96-1.71) |  |

^1^In Burkina Faso, standard of care is VIA/VILI (VI) and is used as reference in relative sensitivity/specificity estimates; ^2^ positive for HC-II (using RLU ≥20) and HPV16 by INNO-LiPA; ^3^ positive for HC-II (using RLU ≥20) and any of HPV16, HPV18 or HPV45 by INNO-LiPA; ^4^ positive for HC-II (using RLU ≥20) and any HPV16/18/45/31/33/35/52/58; ^5^ positive for HC-II (using RLU ≥20) and any HPV16/33/35/58; ^6^calculated among women testing positive for HPV DNA, using HC-II ≥20RLU to define test positive (as sensitivity is equivalent irrespective of threshold used, a higher threshold was used to maximise specificity); ^7^test positive if HPV16 or HPV18 positive, or cytology [ASCUS+] when negative for both HPV16 and HPV18; ^8^test positive if HPV16 or HPV18 positive, or VIA abnormal when negative for both HPV16 and HPV18
