# Supplementary material for: Diagnostic accuracy of cervical cancer screening and screening–triage strategies among women living with HIV-1 in Burkina Faso and South Africa: A cohort study
Source: PLoS Med. 2021 Mar 4;18(3):e1003528. doi: 10.1371/journal.pmed.1003528 (PMC7971880; doi:10.1371/journal.pmed.1003528)
Supplement: S4 Table — (DOCX) [file pmed.1003528.s005.docx]

**S4 Table.** Diagnostic accuracy of screening strategies for detection of **prevalent CIN2+** among 576 unscreened WLHIV in SA

| **Strategy** | **Tests performed, n** | **Test positive (Colposcopies indicated), n** | **CIN2+ identified, n** | **Colposcopies to detect 1 case of CIN2+, n** | **N colpo per 1000 women screened** | **Sensitivity % (95%CI)** | **Specificity (95%CI)** | **PPV (95%CI)** | **1-NPV (95%CI)** | **Sensitivity relative to standard of care^1^** | **Specificity relative to standard of care^1^** |
| --- | --- | --- | --- | --- | --- | --- | --- | --- | --- | --- | --- |
| **Standalone tests** |  |  |  |  |  |  |  |  |  |  |  |
| VIA only | 576 | 162 (28.1) | 58 | 2.8 | 281 | 45.0 (36.2-54.0) | 76.7 (72.5-80.6) | 35.8 (28.4-43.7) | 27.1 (13.6-21.1) | 0.65 (0.53-0.80) | 0.94 (0.89-1.01) |
| VIA or VILI positive (VIA/VILI) | 576 | 239 (41.5) | 81 | 3.0 | 415 | 62.8 (53.8-71.1) | 64.7 (60.0-69.1) | 33.9 (27.9-40.3) | 24.2 (10.7-18.4) | 0.90 (0.76-1.06) | 0.80 (0.74-0.86) |
| Cytology ≥LSIL | 561 | 504 (89.8) | 124 | 4.1 | 898 | 97.6 (93.3-99.5) | 12.4 (9.5-15.9) | 24.6 (20.9-28.6) | 15.3 (1.1-14.6) | 1.39 (1.25-1.56) | 0.15 (0.12-0.19) |
| Cytology ≥HSIL^1^ | 561 | 169 (30.1) | 89 | 1.9 | 301 | 70.1 (61.3-77.9) | 81.6 (77.6-85.1) | 52.7 (44.9-60.4) | 19.7 (7.0-13.1) | 1.00 | 1.00 |
| HC-II (RLU ≥1) | 573 | 342 (59.7) | 112 | 3.1 | 597 | 86.8 (79.7-92.1) | 48.2 (43.5-53.0) | 32.7 (27.8-38.0) | 17.4 (4.3-11.5) | 1.25 (1.11-1.40) | 0.60 (0.54-0.65) |
| HC-II (RLU ≥5) | 573 | 287 (50.1) | 103 | 2.8 | 501 | 79.8 (71.9-86.4) | 58.6 (53.8-63.2) | 35.9 (30.3-41.7) | 19.1 (6.0-13.0) | 1.16 (1.03-1.29) | 0.71 (0.66-0.77) |
| HC-II (RLU ≥10) | 573 | 264(46.1) | 98 | 2.7 | 461 | 76.0 (67.7-83.1) | 62.6 (57.9-67.1) | 37.1 (31.3-43.3) | 20.0 (6.9-13.9) | 1.10 (0.98-1.24) | 0.77 (0.71-0.82) |
| HC-II (RLU ≥20) | 573 | 238 (41.5) | 92 | 2.6 | 415 | 71.3 (62.7-78.9) | 67.1 (62.5-71.5) | 38.7 (32.4-45.2) | 21.0 (7.9-1.9) | 1.04 (0.92-1.17) | 0.82 (0.77-0.87) |
| ***Restricted genotyping*** |  |  |  |  |  |  |  |  |  |  |  |
| HPV16^2^ | 573 | 73 (12.7) | 34 | 2.1 | 127 | 26.4 (19.0-34.8) | 91.2 (88.2-93.7) | 46.6 (34.8-58.6) | 19.0 (15.7-22.7) | 0.38 (0.28-0.51) | 1.12 (1.06-1.18) |
| HPV16/18/45^3^ | 573 | 142 (24.8) | 51 | 2.8 | 247 | 39.5 (31.0-48.5) | 79.5 (75.4-83.2) | 35.9 (28.0-44.4) | 18.1 (14.6-22.1) | 0.57 (0.46-0.72) | 0.98 (0.92-1.04) |
| 8 HR types^4^ | 573 | 297 (51.8) | 104 | 2.9 | 517 | 80.6 (72.7-87.0) | 56.5 (51.8-61.2) | 35.0 (29.6-40.7) | 9.1 (5.9-13.1) | 1.16 (1.02-1.31) | 0.70 (0.64-0.76) |
| HPV16/33/35/58^5^ | 573 | 189 (33.0) | 82 | 2.3 | 329 | 63.6 (54.6-71.9) | 75.9 (71.6-79.8) | 43.4 (36.2-50.8) | 12.2 (9.1-15.9) | 0.91 (0.78-1.06) | 0.93 (0.87-0.99) |
| **Triage of HPV positive women^6^** |  |  |  |  |  |  |  |  |  |  |  |
| VIA only | 342 | 110 (32.2) | 51 | 2.2 | 192 | 45.5 (36.1-55.2) | 74.3 (68.2-79.9) | 46.4 (36.8-56.1) | 26.3 (20.7-32.5) | 0.61 (0.49-0.76) | - |
| VIA or VILI positive (VIA/VILI) | 342 | 163 (47.7) | 72 | 2.3 | 284 | 64.3 (54.7-73.1) | 60.4 (53.8-66.8) | 44.2 (36.4-52.1) | 22.3 (16.5-29.2) | 0.86 (0.72-1.01) | - |
| Cytology ≥LSIL | 333 | 308 (92.5) | 109 | 2.8 | 538 | 98.2 (93.6-99.8) | 10.4 (6.7-15.1) | 35.4 (30.1-41.0) | 8.0 (1.0-26.0) | 1.31 (1.18-1.46) | - |
| Cytology ≥HSIL | 333 | 150 (45.0) | 83 | 1.8 | 363 | 74.8 (65.6-82.5) | 69.8 (63.3-75.8) | 55.3 (47.0-64.4) | 15.3 (10.4-21.3) | - | - |
| HPV16/18+ or other HR-HPV+ AND reflex HSIL+^7^ | 335 | 208 (62.1) | 93 | 2.2 | 370 | 83.8 (75.6-90.1) | 48.7 (41.9-55.4) | 44.7 (37.8-51.7) | 15.2 (8.6-21.5) | 1.12 (1.04-1.20) |  |
| HPV16/18+ or other HR-HPV+ AND reflex VIA^8^ | 342 | 180 (52.6) | 75 | 2.4 | 314 | 67.0 (57.4-75.6) | 54.3 (47.7-60.9) | 41.7 (34.4-49.2) | 22.8 (16.6-30.1) | 0.90 (0.77-11.06) |  |

^1^In South Africa, standard of care used is cytology; HSIL+ is used as reference in relative sensitivity/specificity estimates; ^2^ positive for HC-II (using RLU ≥1) and HPV16 by INNO-LiPA; ^3^ positive for HC-II (using RLU ≥1) and any of HPV16, HPV18 or HPV45 by INNO-LiPA; ^4^ positive for HC-II (using RLU ≥1) and any HPV16/18/45/31/33/35/52/58; ^5^ positive for HC-II (using RLU ≥1) and any HPV16/33/35/58; ^6^calculated among women testing positive for HPV DNA, using HC-II ≥1RLU to define test positive (maximum sensitivity achieved using ≥1RLU to define test positivity in SA); ^7^test positive if HPV16 or HPV18 positive, or cytology [HSIL+] when negative for both HPV16 and HPV18; ^8^test positive if HPV16 or HPV18 positive, or VIA abnormal when negative for both HPV16 and HPV18
