# Supplementary material for: Diagnostic accuracy of cervical cancer screening and screening–triage strategies among women living with HIV-1 in Burkina Faso and South Africa: A cohort study
Source: PLoS Med. 2021 Mar 4;18(3):e1003528. doi: 10.1371/journal.pmed.1003528 (PMC7971880; doi:10.1371/journal.pmed.1003528)
Supplement: S5 Table — (DOCX) [file pmed.1003528.s006.docx]

**S5 Table. Diagnostic accuracy of screening tests (HPV-DNA, VIA and cytology) for CIN2+/CIN3+, by age group**

|  |  |  |  | **CIN2+** | |  | **CIN3+** | |
| --- | --- | --- | --- | --- | --- | --- | --- | --- |
|  | **N women** | **Test positive,**  **n % (95%CI)** | **CIN2+, n (%)** | **Sensitivity**  **(%, 95%CI)** | **Specificity**  **(%, 95%CI)** | **CIN3+, n (%)** | **Sensitivity**  **(%, 95%CI)** | **Specificity**  **(%, 95%CI)** |
| **HPV-DNA (HC-II^1^)** |  |  |  |  |  |  |  |  |
| 25-29 years | 221 | 132 (59.7) | 37 (16.7) | 75.7 (58.8-88.2) | 43.5 (36.2-51.0) | 16 (7.2) | 75.0 (47.6-92.7) | 41.5 (34.6-48.5) |
| 30-34 years | 301 | 165 (54.8) | 45 (15.0) | 91.1 (78.8-97.5) | 51.6 (45.3-57.8) | 18 (6.0) | 88.9 (65.3-98.6) | 47.3 (41.4-53.3) |
| 35-39 years | 274 | 130 (47.4) | 45 (16.4) | 95.6 (84.9-99.5) | 62.0 (55.4-68.3) | 19 (6.9) | 89.5 (66.9-98.7) | 55.7 (49.4-61.9) |
| 40-44 years | 215 | 98 (45.6) | 24 (11.2) | 91.7 (73.0-99.0) | 60.2 (52.9-67.2) | 9 (4.2) | 88.9 (51.8-99.7) | 56.3 (49.2-63.2) |
| 45-50 years | 110 | 46 (41.8) | 10 (9.1) | 90.0 (55.5-99.7) | 63.0 (52.8-72.4) | 4 (3.6) | 100.0 (39.8-100.0) | 60.4 (50.4-69.7) |
|  |  |  |  |  |  |  |  |  |
| **HPV-DNA (8HR)** |  |  |  |  |  |  |  |  |
| 25-29 years | 221 | 111 (50.2) | 37 (16.7) | 73.0 (55.9-86.2) | 54.3 (46.9-61.7) | 16 (7.2) | 75.0 (47.6-92.7) | 51.7 (44.6-58.7) |
| 30-34 years | 301 | 140 (46.5) | 45 (15.0) | 86.7 (73.2-94.9) | 60.5 (54.3-66.6) | 18 (6.0) | 88.9 (65.3-98.6) | 56.2 (50.2-62.0) |
| 35-39 years | 274 | 106 (38.7) | 45 (16.4) | 91.1 (78.8-97.5) | 71.6 (65.3-77.4) | 19 (6.9) | 89.5 (66.9-98.7) | 65.1 (58.9-70.9) |
| 40-44 years | 215 | 81 (37.7) | 24 (11.2) | 79.2 (57.8-92.9) | 67.5 (60.4-74.1) | 9 (4.2) | 88.9 (51.8-99.7) | 64.6 (57.6-71.1) |
| 45-50 years | 110 | 33 (30.0) | 10 (9.1) | 77.8 (40.0-97.2) | 74.3 (64.6-82.4) | 4 (3.6) | 75.0 (19.4-99.4) | 71.7 (62.1-80.0) |
|  |  |  |  |  |  |  |  |  |
| **VIA** |  |  |  |  |  |  |  |  |
| 25-29 years | 222 | 67 (30.1) | 37 (16.7) | 51.4 (34.4-68.1) | 74.1 (67.1-80.2) | 16 (7.2) | 62.5 (35.4-84.8) | 72.3 (65.7-78.3) |
| 30-34 years | 306 | 78 (25.5) | 45 (14.7) | 44.4 (29.6-60.0) | 77.8 (72.2-82.7) | 18 (5.9) | 50.0 (26.0-74.0) | 76.0 (70.7-80.9) |
| 35-39 years | 273 | 61 (22.3) | 45 (16.5) | 44.4 (29.6-60.0) | 82.0 (76.4-86.8) | 19 (7.0) | 63.2 (38.4-83.7) | 80.7 (75.3-85.4) |
| 40-44 years | 217 | 44 (20.3) | 24 (11.1) | 33.3 (15.6-55.3) | 81.3 (75.1-86.6) | 9 (4.1) | 44.4 (13.7-78.8) | 80.8 (74.7-85.9) |
| 45-50 years | 111 | 28 (25.2) | 10 (9.0) | 50.0 (18.7-81.3) | 77.2 (67.8-85.0) | 4 (3.6) | 50.0 (6.8-93.2) | 75.7 (66.5-83.5) |
|  |  |  |  |  |  |  |  |  |
| **Cytology ASCUS+ (BF only)^2^** |  |  |  |  |  |  |  |  |
| 25-29 years | 94 | 22 (23.4) | 2 (2.1) | 50.0 (1.3-98.7) | 77.2 (67.2-85.3) | 0 (0.0) | - | 76.6 |
| 30-34 years | 127 | 36 (28.3) | 4 (3.1) | 100.0 (39.8-100.0) | 74.0 (65.3-81.5) | 2 (1.6) | 100.0 (15.8-100.0) | 72.8 (64.1-80.4) |
| 35-39 years | 131 | 31 (23.7) | 12 (9.2) | 66.7 (34.9-90.1) | 80.7 (72.4-87.3) | 6 (4.6) | 66.7 (22.3-95.7) | 78.4 (70.2-85.3) |
| 40-44 years | 111 | 32 (28.8) | 8 (7.2) | 87.5 (47.3-99.7) | 75.5 (66.3-83.6) | 2 (1.8) | 50.0 (1.3-98.7) | 71.6 (62.1-79.8) |
| 45-50 years | 69 | 16 (23.2) | 4 (5.8) | 75.0 (19.4-99.4) | 80.0 (68.2-88.9) | 1 (1.4) | 100.0 (2.5-100.0) | 77.9 (66.2-87.1) |
|  |  |  |  |  |  |  |  |  |
| **Cytology HSIL+**  **(SA only)^2^** |  |  |  |  |  |  |  |  |
| 25-29 years | 123 | 38 (30.9) | 34 (27.6) | 58.8 (40.7-75.4) | 79.8 (69.9-87.6) | 16 (13.0) | 75.0 (47.6-92.7) | 75.7 (66.5-83.5) |
| 30-34 years | 166 | 55 (33.1) | 41 (24.7) | 70.7 (54.5-83.9) | 79.2 (71.0-85.9) | 16 (9.6) | 75.0 (47.6-92.7) | 71.3 (63.4-78.4) |
| 35-39 years | 135 | 46 (34.1) | 31 (23.0) | 80.6 (62.5-92.5) | 79.8 (70.8-87.0) | 11 (8.1) | 81.8 (48.2-97.7) | 70.2 (61.3-78.0) |
| 40-44 years | 100 | 23 (23.0) | 15 (15.0) | 66.7 (38.4-88.2) | 84.7 (75.3-91.6) | 6 (6.0) | 100.0 (54.1-100.0) | 81.9 (72.6-89.1) |
| 45-50 years | 37 | 7 (18.9) | 6 (16.2) | 83.3 (35.9-99.6) | 93.5 (78.6-99.2) | 3 (8.1) | 100.0 (29.2-100.0) | 88.2 (72.5-96.7) |
| **HC-II positive -> HSIL+ (SA only)^3^** |  |  |  |  |  |  |  |  |
| 25-29 years | 83 | 33 (39.8) | 26 (31.3) | 69.2 (48.2-85.7) | 73.7 (60.3-84.5) | 12 (14.5) | 91.7 (61.5-99.8) | 69.0 (56.9-79.5) |
| 30-34 years | 103 | 51 (49.5) | 73.7 (56.9) | 73.7 (56.9-86.6) | 64.6 (51.8-76.1) | 14 (13.4) | 85.7 (57.2-98.2) | 56.2 (45.3-66.7) |
| 35-39 years | 76 | 38 (50.0) | 29 (38.2) | 79.3 (60.3-92.0) | 68.1 (52.9-80.9) | 9 (11.8) | 77.8 (40.0-97.2) | 53.7 (41.1-66.0) |
| 40-44 years | 56 | 21 (37.5) | 13 (23.2) | 69.2 (38.6-90.9) | 72.1 (56.3-84.7) | 5 (8.9) | 100.0 (47.8-100.0) | 68.6 (54.1-80.9) |
| 45-50 years | 15 | 7 (46.7) | 5 (33.3) | 100.0 (47.8-100.0) | 80.0 (44.4-97.5) | 3 (20.0) | 100.0 (29.2-100.0) | 66.7 (34.9-90.1) |
|  |  |  |  |  |  |  |  |  |
| **HC-II positive -> VIA+^3^** |  |  |  |  |  |  |  |  |
| 25-29 years | 132 | 41 (31.1) | 28 (21.2) | 53.6 (33.9-72.5) | 75.0 (65.6-83.0) | 12 (9.1) | 58.3 (27.7-84.8) | 71.7 (62.7-79.5) |
| 30-34 years | 165 | 54 (32.7) | 41 (24.8) | 48.8 (32.9-64.9) | 72.6 (63.8-80.2) | 16 (9.7) | 56.3 (29.9-80.2) | 69.8 (61.7-77.0) |
| 35-39 years | 130 | 36 (27.7) | 43 (33.1) | 41.9 (27.0-57.9) | 79.3 (69.3-87.3) | 17 (13.1) | 58.8 (32.9-81.6) | 77.0 (68.1-84.4) |
| 40-44 years | 98 | 29 (29.6) | 22 (22.4) | 36.4 (17.2-59.3) | 72.4 (60.9-82.0) | 8 (8.2) | 50.0 (15.7-84.3) | 72.2 (61.8-81.1) |
| 45-50 years | 46 | 15 (32.6) | 9 (19.6) | 44.4 (13.7-78.8) | 70.3 (53.0-84.1) | 4 (8.7) | 50.0 (6.8-93.2) | 69.0 (52.9-82.4) |
|  |  |  |  |  |  |  |  |  |
| **HC-II positive -> HPV16/18 OR HSIL+ ^3^** |  |  |  |  |  |  |  |  |
| 25-29 years | 131 | 59 (45.0) | 28 (21.4) | 78.6 (59.0-91.7) | 64.1 (54.0-73.3) | 12 (9.2) | 100.0 (73.5-100.0) | 60.5 (51.1-69.3) |
| 30-34 years | 160 | 81 (50.6) | 41 (25.6) | 85.4 (70.8-94.4) | 61.3 (52.0-70.1) | 16 (10.0) | 93.8 (69.8-99.8) | 54.2 (45.7-62.5) |
| 35-39 years | 128 | 68 (53.1) | 42 (32.8) | 81.0 (65.9-91.4) | 60.5 (49.3-70.8) | 16 (12.5) | 81.3 (54.4-96.0) | 50.9 (41.3-60.5) |
| 40-44 years | 94 | 51 (54.3) | 21 (22.3) | 66.7 (43.0-85.4) | 49.3 (37.4-61.3) | 7 (7.4) | 85.7 (42.1-99.6) | 48.3 (37.4-59.2) |
| 45-50 years | 42 | 18 (42.9) | 8 (19.0) | 100.0 (63.1-100.0) | 70.6 (52.5-84.9) | 4 (9.5) | 100.0 (39.8-100.0) | 63.2 (46.0-78.2) |
|  |  |  |  |  |  |  |  |  |
| **HC-II positive -> HPV16/18 OR ASCUS+ ^3^** |  |  |  |  |  |  |  |  |
| 25-29 years | 131 | 104 (79.4) | 28 (21.4) | 96.4 (81.7-99.9) | 25.2 (17.2-34.8) | 12 (9.2) | 100.0 (73.5-100.0) | 22.7 (15.5-31.3) |
| 30-34 years | 160 | 132 (82.5) | 41 (25.6) | 100.0 (91.4-100.0) | 23.5 (16.2-32.2) | 16 (10.0) | 100.0 (79.4-100.0) | 19.4 (13.3-26.9) |
| 35-39 years | 128 | 105 (82.0) | 42 (32.8) | 97.6 (87.4-99.9) | 25.6 (16.8-36.1) | 16 (12.5) | 93.8 (69.8-99.8) | 19.6 (12.7-28.2) |
| 40-44 years | 94 | 80 (85.1) | 21 (22.3) | 95.2 (76.2-99.9) | 17.8 (9.8-28.5) | 7 (7.4) | 85.7 (42.1-99.6) | 14.9 (8.2-24.2) |
| 45-50 years | 43 | 26 (60.5) | 9 (20.9) | 100.0 (66.4-100.0) | 50.0 (32.4-67.6) | 4 (9.3) | 100.0 (39.8-100.0) | 43.6 (27.8-60.4) |
|  |  |  |  |  |  |  |  |  |
| **HC-II positive -> HPV16/18 OR VIA+ ^3^** |  |  |  |  |  |  |  |  |
| 25-29 years | 132 | 71 (53.8) | 28 (21.2) | 82.1 (63.1-93.9) | 53.8 (43.8-63.7) | 12 (9.1) | 91.7 (61.5-99.8) | 50.0 (40.7-59.3) |
| 30-34 years | 165 | 83 (50.3) | 41 (24.8) | 73.2 (57.1-85.8) | 57.3 (48.1-66.1) | 16 (9.7) | 81.3 (54.4-96.0) | 53.0 (44.7-61.2) |
| 35-39 years | 130 | 61 (46.9) | 43 (33.1) | 62.8 (46.7-77.0) | 60.9 (49.9-71.2) | 17 (13.1) | 70.6 (44.0-89.7) | 56.6 (47.0-65.9) |
| 40-44 years | 98 | 48 (49.0) | 22 (22.4) | 54.5 (32.2-75.6) | 52.6 (40.8-64.2) | 8 (8.2) | 62.5 (24.5-91.5) | 52.2 (41.4-62.9) |
| 45-50 years | 45 | 22 (48.9) | 8 (17.8) | 75.0 (34.9-96.8) | 56.8 (39.5-72.9) | 4 (8.9) | 75.0 (19.4-99.4) | 53.7 (37.4-69.3) |
|  |  |  |  |  |  |  |  |  |

^1^using 1RLU to determine test positive; ^2^due to differences in diagnostic accuracy of cervical cytology in BF and SA, different cut-off to define test positive were used (ASCUS+ in BF and HSIL+ in SA); PPV=positive predictive value; NPV=negative predictive value (1-NPV is the proportion of false negative among test negative); ^3^triage of HC-II positive women
