# Supplementary material for: Diagnostic accuracy of cervical cancer screening and screening–triage strategies among women living with HIV-1 in Burkina Faso and South Africa: A cohort study
Source: PLoS Med. 2021 Mar 4;18(3):e1003528. doi: 10.1371/journal.pmed.1003528 (PMC7971880; doi:10.1371/journal.pmed.1003528)
Supplement: S6 Table — (DOCX) [file pmed.1003528.s007.docx]

**S6 Table.** Diagnostic accuracy of cervical cancer screening strategies for **CIN3+ detection** among women living with HIV (WLHIV), stratified by ART status

|  |  |  | **CIN3+** | | | | |
| --- | --- | --- | --- | --- | --- | --- | --- |
|  | **N screened** | **Test positive,**  **n (%)** | **N CIN3+ identified** | **Sensitivity (95%CI)** | **Specificity (95%CI)** | **PPV (95%CI)** | **1-NPV (95%CI)** |
| **VIA** |  |  |  |  |  |  |  |
| ART >2 years | 426 | 89 (20.9) | 8 | 42.1 (20.3-66.5) | 80.1 (75.9-83.9) | 9.0 (4.0-16.9) | 3.3 (1.6-5.8) |
| ART ≤2 years | 330 | 86 (26.1) | 18 | 64.3 (44.1-81.4) | 77.5 (72.3-82.1) | 20.9 (12.9-31.0) | 4.1 (2.0-7.4) |
| ART-naïve | 321 | 93 (29.0) | 10 | 55.6 (308-78.5) | 72.6 (67.2-77.6) | 10.8 (5.3-18.9) | 3.5 (1.5-6.8) |
| **VIA/VILI** |  |  |  |  |  |  |  |
| ART >2 years | 426 | 135 (31.7) | 12 | 63.2 (38.4-83.7) | 69.8 (65.1-74.2) | 8.9 (4.7-15.0) | 2.4 (1.0-4.9) |
| ART ≤2 years | 330 | 112 (33.9) | 22 | 78.6 (59.0-91.7) | 70.2 (64.7-75.3) | 19.6 (12.7-28.2) | 2.8 (1.0-5.9) |
| ART-naïve | 321 | 114 (35.5) | 11 | 61.1 (35.7-82.7) | 66.0 (60.4-71.3) | 9.7 (4.9-16.6) | 3.4 (1.4-6.8) |
| **HC-II (≥1RLU)** |  |  |  |  |  |  |  |
| ART >2 years | 425 | 170 (40.0) | 15 | 78.9 (54.4-93.9) | 61.8 (56.9-66.6) | 8.8 (5.0-14.1) | 1.6 (0.4-4.0) |
| ART ≤2 years | 327 | 198 (60.6) | 27 | 96.4 (81.7-99.9) | 42.8 (37.1-48.6) | 13.6 (9.2-19.2) | 0.8 (0.0-4.2) |
| ART-naïve | 318 | 187 (58.8) | 14 | 77.8 (52.3-93.6) | 42.3 (36.7-48.1) | 7.5 (4.2-12.2) | 3.1 (0.8-7.6) |
| **8HR^1^** |  |  |  |  |  |  |  |
| ART >2 years | 423 | 134 (31.7) | 15 | 78.9 (54.4 (93.9) | 70.5 (65.8-74.9) | 11.2 (6.4-17.8) | 1.4 (0.4-3.5) |
| ART ≤2 years | 327 | 170 (52.0) | 26 | 92.9 (76.5-99.1) | 51.8 (46.0-57.6) | 15.3 (10.2-21.6) | 1.3 (0.2-4.5) |
| ART-naïve | 318 | 158 (49.7) | 14 | 77.8 (52.4-93.6) | 52.0 (46.2-57.8) | 8.9 (4.9-14.4) | 2.5 (0.7-6.3) |
| **Cytology ASCUS+ (BF only)** |  |  |  |  |  |  |  |
| ART >2 years | 209 | 41 (19.6) | 2 | 50.0 (6.8-93.2) | 81.0 (74,9-86.1) | 4.9 (0.6-16.5) | 1.2 (0.1-4.2) |
| ART ≤2 years | 162 | 55 (34.0) | 5 | 83.3 (35.9-99.6) | 67.9 (60.0-75.2) | 9.1 (3.0-20.0) | 0.9 (0.0-5.1) |
| ART-naïve | 118 | 33 (28.0) | 0 | - | - | - | - |
| **Cytology HSIL+**  **(SA only)** |  |  |  |  |  |  |  |
| ART >2 years | 201 | 47 (23.4) | 11 | 78.6 (49.2-95.3) | 80.7 (74.4-86.1) | 23.4 (12.3-38.0) | 1.9 (0.4-5.6) |
| ART ≤2 years | 160 | 65 (40.6) | 19 | 95.0 (75.1-99.9) | 67.1 (58.7-74.8) | 29.2 (18.6-41.8) | 1.1 (0.0-5.7) |
| ART-naïve | 192 | 56 (29.2) | 12 | 66.7 (41.0-86.7) | 74.7 (67.6-81.0) | 21.4 (11.6-34.4) | 4.4 (1.6-9.4) |
| **HC-II 🡺 VIA/VILI** |  |  |  |  |  |  |  |
| ART >2 years | 170 | 74 (43.5) | 10 | 66.7 (38.4-88.2) | 58.7 (50.5-66.5) | 13.5 (6.7-23.5) | 5.2 (1.7-11.7) |
| ART ≤2 years | 198 | 83 (41.9) | 21 | 77.8 (57.7-91.4) | 63.7 (56.1-70.9) | 25.3 (16.4-36.0) | 5.2 (1.9-11.0) |
| ART-naïve | 187 | 75 (40.1) | 8 | 57.1 (28.9-82.3) | 61.3 (53.6-68.6) | 10.7 (4.7-19.9) | 5.4 (2.0-11.3) |
| **HC-II 🡺 HSIL+**  **(SA only)** |  |  |  |  |  |  |  |
| ART >2 years | 94 | 38 (40.4) | 9 | 90.0 (55.5-99.7) | 65.5 (54.3-75.5) | 23.7 (11.4-40.2) | 1.8 (0.0-9.6) |
| ART ≤2 years | 120 | 63 (52.5) | 19 | 100.0 (82.4-100.0) | 56.4 (46.2-66.3) | 30.2 (19.2-43.0) | 0.0 (0.0-6.3) |
| ART-naïve | 117 | 48 (41.0) | 10 | 71.4 (41.9-91.6) | 63.1 (53.0-72.4) | 20.8 (10.5-35.0) | 5.8 (1.6-14.2) |

^1^ positive for HC-II (using RLU ≥1) and any of HPV16/18/45/31/33/35/52/58
