# Supplementary material for: Diagnostic accuracy of cervical cancer screening and screening–triage strategies among women living with HIV-1 in Burkina Faso and South Africa: A cohort study
Source: PLoS Med. 2021 Mar 4;18(3):e1003528. doi: 10.1371/journal.pmed.1003528 (PMC7971880; doi:10.1371/journal.pmed.1003528)
Supplement: S7 Table — (DOCX) [file pmed.1003528.s008.docx]

**S7 Table.** Diagnostic accuracy of cervical cancer screening strategies for **CIN2+ detection** among women living with HIV (WLHIV), stratified by ART status

|  | **N screened** | **Test positive** | **N CIN2+ identified** | **No. Colpo per CIN2+** | **N colpo per 1000 women** | **Sensitivity (95%CI)** | **Specificity (95%CI)** | **PPV (95%CI)** | **1-NPV (95%CI)** |
| --- | --- | --- | --- | --- | --- | --- | --- | --- | --- |
| **VIA** |  |  |  |  |  |  |  |  |  |
| ART >2 years | 426 | 89 | 14 | 6.4 | 209 | 31.8 (18.6-47.6) | 80.4 (76.0-84.2) | 15.7 (8.9-25.0) | 8.9 (6.1-12.5) |
| ART ≤2 years | 330 | 86 | 32 | 2.7 | 261 | 53.3 (40.0-66.3) | 80.0 (74.7-84.6) | 37.2 (27.0-48.3) | 11.5 (7.8-16.2) |
| ART-naïve | 321 | 93 | 25 | 3.7 | 290 | 46.3 (32.6-60.4) | 74.5 (68.9-79.6) | 26.9 (18.2-37.1) | 12.7 (8.7-17.8) |
| **VIA/VILI** |  |  |  |  |  |  |  |  |  |
| ART >2 years | 426 | 135 | 23 | 5.9 | 317 | 52.3 (36.7-67.5) | 70.7 (65.8-75.2) | 17.0 (11.1-24.5) | 7.2 (4.5-10.8) |
| ART ≤2 years | 330 | 112 | 44 | 2.5 | 339 | 73.3 (60.3-83.9) | 74.8 (69.2-79.9) | 39.3 (30.2-49.0) | 7.3 (4.3-11.6) |
| ART-naïve | 321 | 114 | 31 | 3.7 | 355 | 57.4 (43.2-70.8) | 68.9 (63.0-74.4) | 27.2 (19.3-36.3) | 11.1 (7.2-16.2) |
| **HC-II (≥1RLU)** |  |  |  |  |  |  |  |  |  |
| ART >2 years | 425 | 170 | 38 | 4.5 | 400 | 86.4 (72.6-94.8) | 65.4 (60.3-70.1) | 22.4 (16.3-29.4) | 2.4 (0.9-5.1) |
| ART ≤2 years | 327 | 198 | 58 | 3.4 | 606 | 96.7 (88.5-99.6) | 47.6 (41.4-53.7) | 29.3 (23.1-36.2) | 1.6 (0.2-5.5) |
| ART-naïve | 318 | 187 | 45 | 4.2 | 594 | 83.3 (70.7-92.1) | 46.2 (40.1-52.4) | 24.1 (18.1-30.8) | 6.9 (3.2-12.6) |
| **8HR** |  |  |  |  |  |  |  |  |  |
| ART >2 years | 423 | 134 | 33 | 4.1 | 317 | 76.7 (61.4-88.2) | 73.4 (68.7-77.8) | 24.6 (17.6-32.8) | 3.5 (1.7-6.3) |
| ART ≤2 years | 327 | 170 | 56 | 3.0 | 520 | 93.3 (83.8-98.2) | 57.3 (51.1-63.3) | 32.9 (25.9-40.6) | 2.5 (0.7-6.4) |
| ART-naïve | 318 | 158 | 42 | 3.8 | 497 | 77.8 (64.4-88.0) | 56.1 (49.8-62.1) | 26.6 (19.9-34.2) | 7.5 (3.9-12.7) |
| **Cytology ASCUS+ (BF only)** |  |  |  |  |  |  |  |  |  |
| ART >2 years | 209 | 41 | 9 | 4.6 | 196 | 75.0 (42.8-94.5) | 83.8 (77.8-88.6) | 22.0 (10.6-37.6) | 1.8 (0.4-5.1) |
| ART ≤2 years | 162 | 55 | 10 | 5.5 | 340 | 83.3 (51.6-97.9) | 70.0 (62.0-77.2) | 18.2 (9.1-30.9) | 1.9 (0.2-6.6) |
| ART-naïve | 118 | 33 | 2 | 16.5 | 280 | 50.0 (6.8-93.2) | 72.8 (63.7-80.7) | 6.1 (0.7-20.2) | 2.4 (0.3-8.2) |
| **Cytology HSIL+**  **(SA only)** |  |  |  |  |  |  |  |  |  |
| ART >2 years | 201 | 47 | 24 | 2.0 | 234 | 77.4 (58.9-90.4) | 86.5 (80.4-91.2) | 51.1 (36.1-65.9) | 95.5 (90.9-98.2) |
| ART ≤2 years | 160 | 65 | 39 | 1.7 | 406 | 84.8 (71.1-93.7) | 77.2 (68.4-84.5) | 60.0 (47.1-72.0) | 92.6 (85.4-97.0) |
| ART-naïve | 192 | 56 | 26 | 2.2 | 292 | 53.1 (38.3-67.5) | 79.0 (71.4-85.4) | 46.4 (33.0-60.3) | 83.1 (75.7-89.0) |
| **HC-II 🡺 VIA/VILI** |  |  |  |  |  |  |  |  |  |
| ART >2 years | 170 | 74 | 21 | 3.5 | 174 | 55.3 (38.3-71.4) | 59.8 (51.0-68.3) | 28.4 (18.5-40.1) | 17.7 (10.7-26.8) |
| ART ≤2 years | 198 | 83 | 42 | 2.0 | 254 | 72.4 (59.1-83.3) | 70.7 (62.4-78.1) | 50.6 (39.4-61.8) | 13.9 (8.2-21.6) |
| ART-naïve | 187 | 75 | 26 | 2.9 | 238 | 57.8 (42.2-72.3) | 65.5 (57.1-73.3) | 34.7 (24.0-46.5) | 17.0 (10.5-25.2) |
| **HC-II 🡺 HSIL+**  **(SA only)** |  |  |  |  |  |  |  |  |  |
| ART >2 years | 94 | 38 | 21 | 1.8 | 191 | 80.8 (60.6-93.4) | 75.0 (63.0-84.7) | 55.3 (38.3-71.4) | 8.9 (3.0-19.6) |
| ART ≤2 years | 120 | 63 | 38 | 1.7 | 396 | 86.4 (72.6-94.8) | 67.1 (55.4-77.5) | 60.3 (47.2-72.4) | 10.5 (4.0-21.5) |
| ART-naïve | 117 | 48 | 24 | 2.0 | 250 | 58.5 (42.1-73.7) | 68.4 (56.7-78.6) | 50.0 (35.2-64.8) | 24.6 (15.1-36.5) |

8HR=positive for HC-II (using RLU ≥1) and any HPV16/18/45/31/33/35/52/58
