# Supplementary material for: Diagnostic accuracy of cervical cancer screening and screening–triage strategies among women living with HIV-1 in Burkina Faso and South Africa: A cohort study
Source: PLoS Med. 2021 Mar 4;18(3):e1003528. doi: 10.1371/journal.pmed.1003528 (PMC7971880; doi:10.1371/journal.pmed.1003528)
Supplement: S8 Table — (DOCX) [file pmed.1003528.s009.docx]

**S8 Table. HPV test positivity at baseline and endline and type-specific HR-HPV infection according to CIN status at baseline and endline among 933 WLHIV followed over 16 months in Burkina Faso and South Africa**

|  | **CIN status at baseline** | **CIN status at endline** | **N (%)** | **HPV+**  **baseline, n (%)** | **HPV+**  **endline, n (%)** | **Type-specific persistence, n (%)*** |
| --- | --- | --- | --- | --- | --- | --- |
|  |  |  | **N=933** | **N=903** | **N=903** | **N=903** |
| Twice ≤CIN1 | ≤CIN1 | ≤CIN1 | 782 | 325 (43.2) | 298 (39.5) | 156 (20.7) |
| Incident CIN2+ | ≤CIN1 | CIN2+ | 27 | 25 (92.6) | 26 (96.3) | 21 (77.8) |
| Underwent management, ≤CIN1 | CIN2+ | ≤CIN1 | 82 | 74 (91.4) | 46 (58.2) | 30 (37.0) |
| Underwent management, CIN2+ redetection | CIN2+ | CIN2+ | 6 | 6 (100.0) | 5 (83.3) | 4 (66.7) |
| No management, CIN2+ redetection | CIN2+ | CIN2+ | 20 | 17 (85.0) | 18 (90.0) | 14 (70.0) |
| No management, spontaneous regression | CIN2+ | ≤CIN1 | 16 | 13 (76.5) | 12 (70.6) | 6 (35.3) |

*positive for the same HR-HPV type at baseline and endline visits; restricted to HC-II positive at baseline for more clinically relevant infections; there were 933 women with matched histology and genotyping at both time points; 903 women also had data for HC-II at baseline and careHPV at endline
