# Supplementary material for: Diagnostic accuracy of cervical cancer screening and screening–triage strategies among women living with HIV-1 in Burkina Faso and South Africa: A cohort study
Source: PLoS Med. 2021 Mar 4;18(3):e1003528. doi: 10.1371/journal.pmed.1003528 (PMC7971880; doi:10.1371/journal.pmed.1003528)
Supplement: S9 Table — (DOCX) [file pmed.1003528.s010.docx]

**S9 Table. CIN2+ incidence at endline among baseline screen negative women, by ART status (countries combined)**

|  | All women  N=27 (3.3%) | | | ART>2 years  N=7 (2.1%) | | | ART<2 years  N=9 (4.2%) | | | ART naïve  N=11 (4.3%) | | |
| --- | --- | --- | --- | --- | --- | --- | --- | --- | --- | --- | --- | --- |
|  | N tested | **Screen negative** | Screen  positive | N tested | **Screen negative** | Screen  positive | N tested | **Screen negative** | Screen positive | N tested | **Screen negative** | Screen positive |
| **CIN2+** |  |  |  |  |  |  |  |  |  |  |  |  |
| VIA | 809 | **14 (2.2)** | 13 (7.6) | 337 | **3 (1.1)** | 4 (6.1) | 214 | **5 (2.9)** | 4 (9.1) | 258 | **6 (3.1)** | 5 (8.1) |
| VIA/VILI | 809 | **11 (1.9)** | 16 (7.0) | 337 | **2 (0.8)** | 5 (5.1) | 214 | **5 (3.2)** | 4 (7.1) | 258 | **4 (2.2)** | 7 (9.2) |
| LSIL+ | 779 | **2 (0.5)** | 25 (6.3) | 322 | **0 (0.0)** | 7 (4.8) | 208 | **2 (2.1)** | 7 (6.2) | 249 | **0 (0.0)** | 11 (8.0) |
| HSIL+ | 779 | **12 (1.7)** | 15 (19.0) | 322 | **2 (0.6)** | 5 (20.0) | 208 | **6 (3.3)** | 3 (12.0) | 249 | **4 (1.8)** | 7 (24.1) |
| HC-II (≥1RLU) | 803 | **2 (0.5)** | 25 (6.9) | 335 | **2 (0.9)** | 5 (4.1) | 212 | **0 (0.0)** | 9 (8.2) | 256 | **0 (0.0)** | 11 (8.5) |
| HC-II (≥20RLU) | 803 | **10 (1.7)** | 17 (7.5) | 337 | **3 (1.1)** | 4 (5.4) | 214 | **3 (2.2)** | 6 (7.7) | 258 | **4 (2.3)** | 7 (8.8) |
| 8 HR | 789 | **3 (0.6)** | 24 (8.5) | 328 | **3 (1.1)** | 4 (7.3) | 210 | **3 (2.1)** | 6 (9.2) | 247 | **4 (2.2)** | 7 (10.9) |

8HR=positive for HC-II (using RLU ≥1) and any of HPV16/18/45/31/33/35/52/58
