# Supplementary material for: Diagnostic accuracy of cervical cancer screening and screening–triage strategies among women living with HIV-1 in Burkina Faso and South Africa: A cohort study
Source: PLoS Med. 2021 Mar 4;18(3):e1003528. doi: 10.1371/journal.pmed.1003528 (PMC7971880; doi:10.1371/journal.pmed.1003528)
Supplement: S10 Table — (DOCX) [file pmed.1003528.s011.docx]

**S10 Table.** Diagnostic accuracy of endline screening strategies for detection of **cumulative CIN2+** prevalence among 431 WLHIV in BF and 415 in SA, excluding women who were treated for baseline CIN2+

| **Strategy** | **Tests performed, n** | **Test positive (Colposcopies indicated), n** | **CIN2+ identified, n** | **Colposcopies to detect 1 case of CIN2+, n** | **N colpo per 1000 women screened** | **Sensitivity % (95%CI)** | **Specificity (95%CI)** | **PPV (95%CI)** | **NPV (95%CI)** | **AUC** |
| --- | --- | --- | --- | --- | --- | --- | --- | --- | --- | --- |
| **Burkina Faso (n=5, 1.2%)** |  |  |  |  |  |  |  |  |  |  |
| VIA positive only | 431 | 52 | 1 | 52.0 | 121 | 20.0 (0.5-71.6) | 88.0 (84.6-91.0) | 1.9 (0.0-10.3) | 98.9 (97.3-99.7) | 0.54 (0.34-0.74) |
| VIA or VILI positive (VI) | 431 | 66 | 2 | 33.0 | 153 | 40.0 (5.3-85.3) | 85.0 (81.2-88.2) | 3.8 (0.4-10.5) | 99.2 (97.6-99.8) | 0.63 (0.38-0.87) |
| Cytology ≥LSIL | 393 | 29 | 1 | 29.0 | 74 | 33.3 (0.8-90.6) | 92.8 (89.8-95.2) | 3.5 (0.1-17.8) | 99.5 (98.0-99.9) | 0.63 (0.30-0.96) |
| Cytology ≥HSIL | 393 | 5 | 0 | - | 13 | 0.0 (0.0-70.8) | 98.7 (97.0-99.6) | 0.0 (0.0-52.2) | 99.2 (97.8-99.8) | 0.49 (0.49-0.50) |
| CareHPV | 403 | 175 | 5 | 35.0 | 434 | 100.0 (47.8-100.0) | 57.3 (52.3-62.2) | 2.9 (0.9-6.5) | 100.0 (98.4-100.0) | 0.79 (0.76-0.81) |
|  |  |  |  |  |  |  |  |  |  |  |
| **South Africa (n=42, 10.0%)** |  |  |  |  |  |  |  |  |  |  |
| VIA only | 415 | 80 | 12 | 6.7 | 193 | 28.6 (15.7-44.6) | 81.8 (77.5-85.6) | 15.0 (8.0-24.7) | 91.0 (87.5-93.9) | 0.55 (0.48-0.62) |
| VIA or VILI positive (VI) | 415 | 184 | 27 | 6.8 | 443 | 64.3 (48.0-78.4) | 57.9 (52.7-63.0) | 14.7 (9.9-20.6) | 93.5 (89.5-96.3) | 0.61 (0.53-0.69) |
| Cytology ≥LSIL | 412 | 331 | 38 | 8.7 | 804 | 90.5 (77.4-97.3) | 20.8 (16.8-25.3) | 11.5 (8.3-15.4) | 95.1 (87.8-98.6) | 0.56 (0.51-0.61) |
| Cytology ≥HSIL | 412 | 71 | 27 | 2.7 | 172 | 64.3 (48.0-78.4) | 88.1 (84.4-91.2) | 38.0 (26.8-50.3) | 95.6 (92.8-97.5) | 0.76 (0.69-0.84) |
| CareHPV | 415 | 179 | 39 | 4.6 | 431 | 92.9 (80.5-98.5) | 62.5 (57.3-67.4) | 21.8 (16.0-28.6) | 98.7 (96.3-99.7) | 0.78 (0.73-0.82) |
|  |  |  |  |  |  |  |  |  |  |  |
| **Sites combined (n=47, 5.6%)** |  |  |  |  |  |  |  |  |  |  |
| VIA positive only | 846 | 132 | 13 | 10.2 | 156 | 27.7 (15.6-42.6) | 85.1 (82.4-87.5) | 9.9 (5.4-16.3) | 95.2 (93.4-96.7) | 0.56 (0.50-0.63) |
| VIA or VILI positive (VI) | 846 | 250 | 29 | 8.6 | 296 | 61.7 (46.4-75.5) | 72.3 (69.1-75.4) | 11.6 (7.9-16.2) | 97.0 (95.3-98.2) | 0.67 (0.60-0.74) |
| Cytology ≥LSIL | 805 | 360 | 39 | 9.2 | 447 | 86.7 (73.2-94.9) | 57.8 (54.2-61.3) | 10.8 (7.8-14.5) | 98.7 (97.1-99.5) | 0.72 (0.67-0.78) |
| Cytology ≥HSIL | 805 | 76 | 27 | 2.8 | 94 | 60.0 (44.3-74.3) | 93.6 (91.6-95.2) | 35.5 (24.9-47.3) | 97.5 (96.1-98.5) | 0.77 (0.70-0.84) |
| CareHPV | 818 | 354 | 44 | 8.0 | 433 | 93.6 (82.5-98.7) | 59.8 (56.2-63.3) | 12.4 (9.2-16.3) | 99.4 (98.1-99.9) | 0.77 (0.73-0.81) |
