# Supplementary material for: Diagnostic accuracy of cervical cancer screening and screening–triage strategies among women living with HIV-1 in Burkina Faso and South Africa: A cohort study
Source: PLoS Med. 2021 Mar 4;18(3):e1003528. doi: 10.1371/journal.pmed.1003528 (PMC7971880; doi:10.1371/journal.pmed.1003528)
Supplement: S1 Text — (PDF) [file pmed.1003528.s012.pdf]

Evaluation and impact of screening and treatment approaches for the prevention of cervical neoplasia in HIV-positive women in Burkina Faso and South Africa: HPV in Africa Research Partnership) HARP study

EC Grant Agreement Number: 265396

Deliverable 5.1 Plan of Analysis – Study 1

**Author**	LSHTM Data Management Team
**Submission Date**	
**Reporting period**	1st November 2010 to 30th October 2013
**Document version**	0.1

Table of Contents

Reportable objectives for Deliverable 5.1 Plan of Analysis for Study 1 Report 3

STUDY 1 OBJECTIVES 3

Study 1/Objective 1 3

Study 1/Objective 2 4

Study 1/Objective 3 4

DUMMY TABLES 7

LIST OF APPENDICES

Appendix 1 Performance of cervical cancer screening tests in HIV-positive women in Africa: influence of CD4 counts (Paper 3) 7

Appendix 2 Importance of virological and immunological control on prevalence of HPV infection and CIN2+ among HIV infected African women, taking ART or not (Paper 1&2a) 11

Appendix 3 Comparison of the Digene HC2 HPV DNA Assay with the INNO-LiPA HPV Genotyping Assay for Cervical Cancer Screening in African Women Infected with HIV-1 – HARP study (Paper 4a) 18

Reportable objectives for Deliverable 5.1 Plan of Analysis for Study 1 Report

Plan of analysis for Study 1: Analysis plan to include aims and objectives of the studies, the statistical methods to be used for each analysis, and dummy tables and figures to detail how results will be presented.

This document details the **plans of analysis for Study 1 only**.

STUDY 1 OBJECTIVES

To determine, among HIV-infected African women attending HIV care settings:

1) The relative diagnostic performance of a novel high-risk (HR) HPV DNA rapid test (*CareHPV*) versus other screening tests (cytology, VIA/VILI) to detect high-grade cervical intraepithelial neoplasia (CIN2+). A secondary analysis will evaluate the impact on CIN3+.
2) The prevalence of cervical HPV infection, genotype distribution and associations with cytological and histological cervical lesions, according to HIV-related factors (plasma viral load, CD4+ counts) and exposure to antiretroviral treatment (ART).
3) The diagnostic accuracy of *CareHPV* to detect specific HR-HPV genotypes (demonstrated by HPV genotyping).

Study 1/Objective 1

To answer **Study 1/Objective 1,** we will estimate the positive predictive values (PPV) to detect CIN2+ at baseline by the proportion of participants with positive *CareHPV* test who have CIN-2+; and at follow-up by the proportion of participants with repeat *CareHPV* positive test who develop CIN2+ over follow-up. Similarly, we will estimate the negative predictive values (NPV) by the proportion of participants with negative *CareHPV* test who do not have CIN2+; and at follow-up by the proportion of participants with repeat *CareHPV* negative test who did not develop CIN2+ over follow-up. We will obtain 95% confidence interval (CI) for the PPV and NPV assuming binomial distributions. Subgroup analyses with CIN3+ as an endpoint (only descriptive, as probably little power) will also be done.

Paper 3, with working title “Performance of cervical cancer screening tests in HIV-positive women in Africa: influence of CD4 counts” is in draft manuscript stage at the time of this report. Refer to **Appendix 1** for dummy tables for this paper.

As previously reported in Month 18 report, CareHPV assay has been substituted for Digene HC2 assay in Study 1 (resulting from unavailability of CareHPV kits from the manufacturer).

Therefore Study 1/Objective 1 and its associated analyses and publications will be performed using Digene HC2 data.

Study 1/Objective 2

To answer **Study 1/Objective 2,** we will calculate the prevalence of cervical HPV infection and the genotype distribution by site, and within categories of the factors mentioned, and compares prevalence using odds ratios and 95%CI estimated with logistic regression.

Paper 1/2a, with working title "Importance of virological and immunological control on prevalence of HPV infection and CIN2+ among HIV infected African women, taking ART or not" is in draft manuscript stage at the time of this report. Refer to Appendix 2 for dummy tables for this paper.

Study 1/Objective 3

To answer **Study 1/Objective 3,** we will estimate the sensitivity and specificity and 95%CIs, assuming binomial distributions, of CareHPV compared to results of HPV genotyping using the molecular assay results for HR-HPV detection, for those HR types that CareHPV is supposed to detect.

As previously reported in Month 18 report, CareHPV assay has been substituted for Digene HC2 assay in Study 1 (resulting from unavailability of CareHPV kits from the manufacturer). Therefore Study 1/Objective 3 and its associated analyses and publications will be performed using Digene HC2 data.

Paper 4a, with working title "Comparison of the Digene HC2 HPV DNA Assay with the INNO-LiPA HPV Genotyping Assay for Cervical Cancer Screening in African Women Infected with HIV-1 – HARP study" is in draft manuscript stage at the time of this report. Refer to Appendix 3 for dummy tables for this paper.

In addition to above key analyses and publications related to the key study objectives for Study 1, the HARP study team has generated a list of further analyses for publication, led by team members from the four institutions. This list is summarised in the table below.

	Paper Title	Related to:	Brief Description	Analysis
**1&2a**	**BASELINE PAPER: STUDY DESIGN & CIN ENDPOINT**<br>**&**<br>**EPIDEMIOLOGY OF HPV**	Study 1/<br>Objective 2	1. **HARP Study Design + study population (brief)**<br><br>2. **Epidemiology of CIN:**<br>Prevalence<br>Association with HPV types<br>Association of HIV-related factors<br><br>3. **Epidemiology of hr-HPV infection**<br>Prevalence<br>RF for HPV<br>Association of HIV-related factors	1. Table population characteristics + flowcharts enrolment<br><br>2. CIN prevalence, association with SIL, hrHPV, with CD4/PVL/ART<br>Uni and multivariate<br><br>3. HPV prevalence (by InnoLipa), genotype distribution, association with SIL, with CIN, with other STIs, with CD4/PVL/ART<br>Uni and multivariate RF analyses
**2b**	**EPIDEMIOLOGY OF LR-HPV**	Study 1/<br>Objective 2	**Epidemiology of lr-HPV infection** and **anogenital warts (AGW)** among HIV-infected women<br>Prevalence lr-HPV<br>Prevalence AGW<br>Risk factors for HPV-6 & -11<br>Risk factors for AGW<br>Associations AGW/lr-HPV	Analyse lr-HPV on their own and with any type
**3**	**PERFORMANCE OF SCREENING TO DETECT PREVALENT CIN2+**	Study 1/<br>Objective 1	**Performance of HPV DNA testing and other screening method**s (cytology, VIA/VILI) to **detect CIN2+** of the various screening tests against the gold standard definition of **biopsy** among all screened HIV-positive women.<br>Single test<br>Combination of tests	Standard Sen/Spe/PPV/NPV + Youden or Kappa stats + 95%CI<br><br>Repeat analyses for CIN3<br>Contrast to Firnharber (cf infra)<br><br>Add age effect
**4a**	**PERFORMANCE OF HPV TESTS TO DETECT HPV GENOTYPES**	Study 1/<br>Objective 3	**Performance of Digene HC2** to detect the corresponding targeted hr-HPV types by genotyping<br><br>Comparison of HC2 and InnoLiPA to detect CIN2+	Sen/Spe/PPVNPV + 95%CI<br>v genotyping as gold standard<br><br>And both tests vs CIN2+ as gold standard
**4b**	**COMPARISON OF CAREHPV AND HYBRID CAPTURE 2 ASSAYS FOR DETECTION OF HIGH-RISK HPV DNA IN CERVICAL SAMPLES FROM HIV-1-INFECTED AFRICAN WOMEN**	Supplementary study/<br>Objective 1	**Comparison and concordance of CareHPV vs Digene**<br><br>Published (Online ahead of print in October 2013) in the Journal of Clinical Microbiology	Prevalence of HPV<br>Concordance and kappa statistics<br><br>Discordant cases resolved by INNOLiPA<br>Use RLU levels

	Paper Title	Related to:	Brief Description	Analysis
**5a**	**CLINICAL –EPIDEMIOLOGY OF STI**	Study 1	**Prevalence and Risk factors for MG**<br><br>**Prevalence and RF for cervical infections (NG/CT)**	Site specific but can be contrasted in same paper and analyses combined, eg for influence of HIV-related factors
		Study 1	**Prevalence and Risk factors for vaginal infections (TV, BV, CA)**<br>**BV and vaginal infections as risk factors for CIN and HPV**	Site specific but can be contrasted in same paper and analyses combined, eg for influence of PVL, CD4, ART
**6**	**ROLE OF COLPO**	Study 1 + 2	Contrasting colpo impression/diagnosis (ie HG vs LG) vs Swede score vs CIN<br>Combination cyto-colpo vs CIN (part of performance paper)<br>4-quadrant analysis:<br>Does 4-Q add to the diagnosis (vs directed colpo)<br>Detection rate of symptomatic/pathological vs asymptomatic quadrants<br>(additionally, but can overlap with Performance paper does an algo based on HPV/VIA/cyto+4Q bx add to VIA/cyto driven colpo+directed bx)	Stratification by site, by CD4, by ART status
**7a**	**HEALTH ECONOMICS**<br>South Africa	Study 3/ Objective 1, part of 2	**Costing and cost-effectiveness** in terms of:<br>**Incremental costs** of implementing screening and treatment: provision of screening and pre-cancer treatment **within existing HIV services** as well as local costs of cancer treatment.<br>**Incremental cost per case CIN2+ detected** of different screening strategies (VIA, careHPV and cytology): combining costing data with the epidemiological data in a within trial analysis	Costs (range) sensitivity analysis<br><br>Non-parametric bootstrap cost per case detected 95% CI.
**7b**	**HEALTH ECONOMICS**<br>Burkina Faso	Study 3/ Objective 1, part of 2	**Costing and cost-effectiveness** in terms of:<br>**Incremental costs** of implementing screening and treatment: provision of screening and pre-cancer treatment **within existing HIV services** as well as local costs of cancer treatment.<br>**Incremental cost per case CIN2+ detected** of different screening strategies (VIA, careHPV and cytology): combining costing data with the epidemiological data in a within trial analysis	Costs (range) sensitivity analysis<br><br>Non-parametric bootstrap cost per case detected 95% CI

DUMMY TABLES

Appendix 1 Performance of cervical cancer screening tests in HIV-positive women in Africa: influence of CD4 counts (Paper 3)

Table 1. Characteristics of study population

	Burkina Faso (N=629) n (%) or median (IQR)	South Africa (N=625) n (%) or median (IQR)
Age (years)		
Married		
Primary education or less		
≥5 lifetime sexual partners		
Ever used hormonal contraception		
Median no. of pregnancies		
Ever smoked		
Prior history of cervical cancer screening		
Pap smear		
VIA		
Previous treatment of cervical abnormalities		
ART (%)		
CD4+ count, cells/µl (IQR)		
HIV RNA, copies/ml		
HIV disease status		
No ART		
ART, not virally suppressed		
ART and VL undetectable (%)		

Table 2. Proportion of HIV positive women who tested positive, by test and country

Test outcome	Burkina Faso	South Africa
VIA/VILI		
VIA only positive		
VILI only positive		
VIA or VILI positive		
Cytology results		
Normal		
ASCUS		
ASC-H		
LSIL	113 (18.8%)	360 (59.3%)
HSIL	32 (5.3%)	179 (29.5%)
ICC	1 (0.2%)	2 (0.3%)
HR-HPV DNA (HC-2) + (%)	267 (43.8%)	370 (60.0%)

Fig. 1 Study Flow Chart

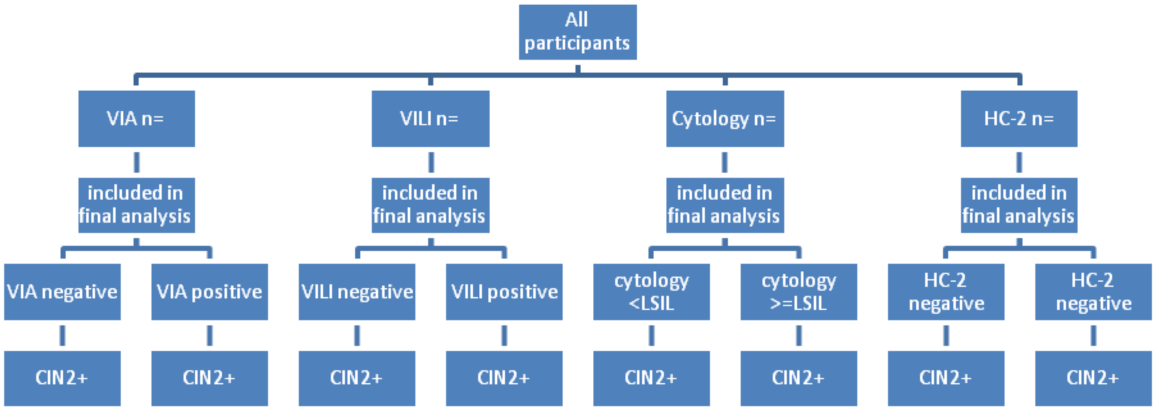

Table 3. Performance and clinical outcomes of different cervical cancer screening strategies for CIN2+ detection in HIV positive women (by CD4+ count stratum) in South Africa and Burkina Faso -

Strategy	Tests performed, n	Colposcopies performed, n	CIN2+ identified, n	Colposcopies to detect 1 case of CIN2+, n	Sensitivity %	Sensitivity relative to standard of care*	False positive rate, %	Specificity relative to standard of care*	Specificity %
**South Africa**									
VIA only									
VIA or VILI positive									
Cytology ≥LSIL									
Cytology ≥HSIL									
HR-HPV									
≥LSIL & HC2									
≥HSIL & HC2									
VI & HC2									
**Burkina Faso**									
VIA only									
VIA or VILI positive									
Cytology ≥LSIL									
Cytology ≥HSIL									
HR-HPV									
≥LSIL & HC2									
≥HSIL & HC2									
VI & HC2									

Table 4. Performance and clinical outcomes of different cervical cancer screening strategies for CIN3+ detection in HIV positive women (by CD4+ count stratum) in South Africa and Burkina Faso (SA: 11, 14, 27 CIN3+ and BF: 3, 2, 8 CIN8+ by cd4 group)

Strategy	Tests performed, n	Colposcopies performed, n	CIN3+ identified, n	Colposcopies to detect 1 case of CIN3+, n	Sensitivity %	Sensitivity relative to standard of care*	False positive rate, %	Specificity relative to standard of care*	Specificity %
**South Africa**									
VIA only									
VIA or VILI positive									
Cytology ≥LSIL									
Cytology ≥HSIL									
HR-HPV									
≥LSIL & HC2									
≥HSIL & HC2									
VI & HC2									
**Burkina Faso**									
VIA only									
VIA or VILI positive									
Cytology ≥LSIL									
Cytology ≥HSIL									
HR-HPV									
≥LSIL & HC2									
≥HSIL & HC2									
VI & HC2									

Appendix 2 Importance of virological and immunological control on prevalence of HPV infection and CIN2+ among HIV infected African women, taking ART or not (Paper 1&2a)

Table 1 Prevalence of hr-HPV

	Burkina Faso						South Africa					
	All participants		<CIN2		CIN2+		All participants		<CIN2		CIN2+	
N												
	n	%	n	%	n	%	n	%	n	%	n	%
Total tested												
Valid genotyping results												
HPV DNA negative												
HPV DNA positive												
lrHPV only												
Any hrHPV												
Single hrHPV												
Multiple hrHPV												
HPV16 or 18												
Nonavalent types												

Table 2a - **hrHPV associated with duration on ART & CD4 in Burkina Faso**

		**non ART users**			**ART users (duration)**								
					**<6 months**			**6-24 months**			**>24 months**		
		**N**	n	%	**N**	n	%	**N**	n	%	**N**	n	%
**CD4**	**<200**												
	**201-350**												
	**351-499**												
	**500+**												
	**Total**												
	**p-value for trend**												

Table 2b - hrHPV associated with duration on ART & CD4 in **South Africa**

		**non ART**			**ART users (duration)**								
					**<6 months**			**6-24 months**			**>24 months**		
		**N**	n	%	**N**	n	%	**N**	n	%	**N**	n	%
**CD4**	**<200**												
	**201-350**												
	**351-499**												
	**500+**												
	**Total**												
	**p-trend**												

Table 3a– hrHPV associated with CD4 according to ART status and PVL in Burkina Faso

		Non ART									ART								
	**PVL**	**< 1000 c/ml**			**1000-9999 c/ml**			**≥ 10,000 c/ml**			**< 1000 c/ml**			**1000-9999 c/ml**			**≥ 10,000 c/ml**		
		**N**	n	%	**N**	n	%	**N**	n	%	**N**	n	%	**N**	n	%	**N**	n	%
**CD4**	**<200**																		
	**201-350**																		
	**351-499**																		
	**500+**																		
	**Total**<br>**p-for trend**																		
	**≤350**																		
	**>350**																		
	**Total**																		

Table 3b – hrHPV associated with CD4 according to ART status and PVL in **South Africa**

			**NON ART**								**ART**								
	**PVL**	**< 1000 c/ml**			**1000-9999 c/ml**			**≥ 10,000 c/ml**			**< 1000 c/ml**			**1000-9999 c/ml**			**≥ 10,000 c/ml**		
		**N**	n	%	**N**	n	%	**N**	n	%	**N**	n	%	**N**	n	%	**N**	n	%
**CD4**	**<200**																		
	**201-350**																		
	**351-499**																		
	**500+**																		
	**Total**<br>**p-trend**																		
	**≤350**																		
	**>350**																		

Table 4a - **CIN2+ associated with duration on ART & CD4 in Burkina Faso**

		**non ART users**			**ART users (duration)**								
					**<6 months**			**6-24 months**			**≥24 months**		
		**N**	n	%	**N**	n	%	**N**	n	%	**N**	n	%
**CD4**	**<200**												
	**201-350**												
	**351-499**												
	**500+**												
	**Total**												
	**p-value for trend**												

Table 4b - CIN2+associated with duration on ART & CD4 in **South Africa**

		**non ART**			**ART users (duration)**								
					**<6 months**			**6-24 months**			**≥24 months**		
		**N**	n	%	**N**	n	%	**N**	n	%	**N**	n	%
**CD4**	**<200**												
	**201-350**												
	**351-499**												
	**500+**												
	**Total**												
	**p-trend**												

Table 5a– **CIN2+associated with CD4 according to ART status and PVL in Burkina Faso**

		Non ART									ART								
	**PVL**	**< 1000 c/ml**			**1000-9999 c/ml**			**≥ 10,000 c/ml**			**< 1000 c/ml**			**1000-9999 c/ml**			**≥ 10,000 c/ml**		
		**N**	n	%	**N**	n	%	**N**	n	%	**N**	n	%	**N**	n	%	**N**	n	%
**CD4**	**<200**																		
	**201-350**																		
	**351-499**																		
	**500+**																		
	**Total**<br>**p-for trend**																		
	**≤350**																		
	**>350**																		
	**Total**																		

Table 5b – CIN2+ associated with CD4 according to ART status and PVL in **South Africa**

			NON ART								ART								
	**PVL**	**< 1000 c/ml**			**1000-9999 c/ml**			**≥ 10,000 c/ml**			**< 1000 c/ml**			**1000-9999 c/ml**			**≥ 10,000 c/ml**		
		**N**	n	%	**N**	n	%	**N**	n	%	**N**	n	%	**N**	n	%	**N**	n	%
**CD4**	**<200**																		
	**201-350**																		
	**351-499**																		
	**500+**																		
	**Total**																		
	**p-trend**																		
	**≤350**																		
	**>350**																		

Table 7 Association of hrHPV infection with sociodemographic HIV-related factors, clinical symptoms and signs and STI, multivariate analysis

	Burkina Faso				South Africa			
	hrHPV infection							
	Count	AOR	95% C.I.	P-value	Count	AOR	95% C.I.	P-value
**Age group at enrolment**								
**25-29**								
**30-34**								
**35-39**								
**40-44**								
**45-50**								
**Ever smoked**								
**never**								
**ever**								
**CD4 count at baseline**								
**>500**								
**351-500**								
**200-350**								
**<200**								
**log10 plasma viral load**								
**<1000**								
**1000-9999**								
**>=10000**								
**Cervical contact bleeding**								
**no**								
**yes**								
**Genital ulcer syndrome**								
**no**								
**yes**								
**B vaginosis**								
**negative**								
**positive**								

Appendix 3 Comparison of the Digene HC2 HPV DNA Assay with the INNO-LiPA HPV Genotyping Assay for Cervical Cancer Screening in African Women Infected with HIV-1 – HARP study (Paper 4a)

TABLE 1. Agreement between the Hybrid Capture-2 (HC2) HPV DNA assay and the INNO-LiPA HPV Genotyping Extra® assay for the detection of 13 high-risk HPV genotypes

	N	INNO-LiPA Positive<br>n (%)	INNO-LiPA Negative<br>n (%)	% agreement	Kappa	P-value
All samples<br>HC2+<br>HC2 -						
<CIN2<br>HC2+<br>HC2 -						
CIN2+<br>HC2+<br>HC2 -						

TABLE 2. Detection rate of the 13 high-risk HPV types by Hybrid Capture-2 (HC2) HPV DNA assay among samples with single HPV infection as identified by INNO-LiPA.

HPV types	INNO-LiPA	HC2	
	n	n	%
HPV 16			
HPV 18			
HPV 31			
HPV 33			
HPV 35			
HPV 39			
HPV 45			
HPV 51			
HPV 52			
HPV 56			
HPV 58			
HPV 59			
HPV 68			
Subtotal hr-HPV			

TABLE 3. Prevalence of high-risk HPV (13 types), as determined by the Hybrid Capture-2 (HC2) HPV DNA assay and the INNO-LiPA HPV genotyping Extra® assay, according to the cytological findings.

	Cytology		High-risk HPV Prevalence HC2		INNO-LiPA	
	n	%	n	%	n	%
Burkina Faso						
Normal						
ASC-US						
LSIL						
ASC-H						
HSIL						
Total						
South Africa						
Nomal						
ASC-US						
LSIL						
ASC-H						
HSIL						
Total						

Alternative table 3 – combining ASCUS & LSIL and ASH-H & HSIL

	Cytology		HR-HPV Prevalence HC2		INNO-LiPA	
	n	%	n	%	n	%
<u>Burkina Faso</u><br>Normal<br>ASC-US/LSIL<br>ASC-H/HSIL<br>Total						
<u>South Africa</u><br>Normal<br>ASC-US/LSIL<br>ASC-H/HSIL<br>Total						

Alternative Table 3 (MS)

	hr-HPV prevalence					
	HC2			INNO-LIPA		
Cytology	Overall	B.F	S.A	Overall	B.F	S.A
Normal						
ASCUS						
LSIL						
HSIL/ASC-H						

TABLE 4. Sensitivity, specificity positive and negative predictive values of the Digene HC2 HPV DNA assay and the INNO-LiPA HPV genotyping Extra assay for the diagnosis of cervical intraepithelial CIN2+ and CIN3+ lesions.

	CIN2+ (n=157)			CIN3+ (n=66)		
Parameters	HC2 RLU>1.0	HC2 RLU>2.0	INNO-LiPA	HC2 RLU>1.0	HC2 RLU>2.0	INNO-LiPA
No. of cases missed by test						
No. testing positive						
Sensitivity (%)						
Specificity (%)						
PPV						
NPV						
