# Supplementary material for: Diagnostic accuracy of cervical cancer screening and screening–triage strategies among women living with HIV-1 in Burkina Faso and South Africa: A cohort study
Source: PLoS Med. 2021 Mar 4;18(3):e1003528. doi: 10.1371/journal.pmed.1003528 (PMC7971880; doi:10.1371/journal.pmed.1003528)
Supplement: Attachment — Submitted filename: Letter of response_Reviewers_211020.docx [file pmed.1003528.s013.docx]

**Re: PMEDICINE-D-20-04079R1: Defining optimal cervical cancer screen and triage strategies for women living with HIV-1 in Sub-Saharan Africa**

21 October 2020

To the Editor of PLOS Medicine,

We wish to submit a revised manuscript and this response letter to Reviewers' comments for the manuscript “**Defining optimal cervical cancer screen and triage strategies for women living with HIV-1 in Sub-Saharan Africa”** (reference number: **PMEDICINE-D-20-04079R1**).

First of all, we would like to thank the reviewers for their valuable comments. Please see below the response to their comments. [Our responses are highlighted in blue.]

Please note that when we discuss revisions made to the manuscript, the location of the revision is indicated by line number in the track mode version of the manuscript.

Thank you for your consideration of this revised manuscript,

Yours sincerely,

Helen Kelly, on behalf of all co-authors

Comments from the reviewers:

**Reviewer #1:** This is an interesting diagnostic accuracy study on cervical cancer screening and triage strategies for women living with HIV-1 in Sub-Saharan Africa. However, there are quite a few issues needing attention.

**1)** The title of "Defining optimal cervical cancer screen and triage strategies..." is a bit misleading. This is basically an observational studies on the diagnostic accuracies of a range of cervical cancer tests on the two cohorts for BF and SA. To develop and validate a diagnostics test, needs to develop/establish a test in a training set and then validate the test in the independent/external validation set, the performance of the diagnostic test needs to be evaluated for both discrimination and calibration. So far, we didn't see any of the above in the paper, therefore it's mainly an observational study reporting on the test results of a range of diagnostic tests.

**Response**: The Reviewer is correct that this study is a prospective observational study designed to evaluate the diagnostic accuracy of cervical cancer screening strategies among women attending routine HIV care in Africa. We are evaluating strategies in different settings, not attempting to validate a diagnostic test *per se*. The performance of each test will vary according to the population being tested. As such, we agree to change title to: “Diagnostic accuracy of cervical cancer screening and screening-triage strategies among women living with HIV-1 in Africa”

**2A).** As this is a diagnostic study, it's not clear what is the gold standard of all the tests. Also, it could be better structured and written in a way - primary outcome, secondary outcome, incremental test accuracies of test 1, 2 and 3. Now it's all over the place.

**Response:** We apologise for the confusion. The gold standard, or reference method, is histology-verified CIN2+/CIN3+. In the final paragraph of the Introduction, we have clarified the index test and reference method used. We also state the primary and secondary objectives of the study (starting line 45):

*The primary objective of* *the current study was to evaluate the diagnostic accuracy of three screening approaches (index tests): HR-HPV DNA tests (Hybrid Capture-II and CareHPV), visual inspection (standard-of-care in BF) and cervical cytology (standard-of-care in SA) for the detection of prevalent CIN2+/CIN3+ (reference method) in screening and in triage (Analysis 1). Secondary objectives were to evaluate the diagnostic accuracy of those test strategies by ART status (Analysis 2). To inform on frequency of screening, we evaluated CIN2+ incidence over 16 months among baseline screen-negative WLHIV (Analysis 3).*

In Methods, line 124, we also define the index tests and the reference method:

*“In the analysis of diagnostic accuracy (****Analysis 1****), the index tests evaluated included VIA alone, VIA/VILI (co-testing when either test is positive), cytology (using thresholds of low-grade squamous intraepithelial lesion, or greater [LSIL+] and high-grade squamous intraepithelial lesion, or greater [HSIL+]) and HR-HPV DNA (Digene HC-II) for the detection of histology-confirmed CIN2+ and CIN3+ (reference method) at baseline.”*

**2B).** What exactly is the research question?

**Response**: We apologise if this is unclear. We point out here in the introduction, penultimate paragraph (line 35), we propose a problem statement *“… cervical cancer screening approaches more commonly utilised in LMIC, including visual inspection methods and cervical cytology have poor sensitivity and specificity for CIN2+ detection and can lack reproducibility in both women in the general population and among WLHIV. Screening and screening-triage strategies that can be easily implemented but with high accuracy to detect CIN2+ are needed*” and in the final paragraph, we state the aim (line 45): “*In .. we report the diagnostic accuracy of HR-HPV DNA tests (Hybrid Capture-II and CareHPV) compared to standard-of-care screening strategies (visual inspection in BF and cervical cytology in SA) for the detection of prevalent CIN2+/CIN3+…”*

**2C).** What's the rationale and link of involving two cohorts from BF and SA? It's like two separate studies.

**Response**: As stated in the first paragraph of the Discussion (line 420), Burkina Faso and South Africa represent contrasting situations of HIV and HPV-related disease in Sub-Saharan Africa. South Africa has both high prevalence of HIV among women aged 15-49 years and high incidence of ICC while Burkina Faso has low HIV prevalence in women and ICC incidence. Both countries have differing modalities for cervical cancer screening. This allows findings to be made generalizable to populations of WLHIV in different settings in low- and middle-income settings.

As requested by other Reviewers and where appropriate, we have combined the findings on diagnostic accuracy for the two countries in the main manuscript (but kept the country stratified analyses in the supplementary information). However, due to the variability in performance of cervical cytology, linked to differences in infrastructure available, and possibly disease prevalence in the two countries (South Africa has well established infrastructure to conduct high quality cytology and higher disease prevalence), we presented diagnostic accuracy of cytology separately by country.

**2D).** Also, what's the point of baseline and endline measurements? For the diagnostic studies, it is 'on the spot' test which is very different from prognostic test with follow-ups. Basically the follow-ups in the study didn't contribute to the evaluation of the tests.

**Response**: The baseline study data were used to evaluate diagnostic accuracy of different screening or screening-triage approaches and to understand which approach had the best combined sensitivity and specificity to detect CIN2+/CIN3+ at one time point in a population of largely unscreened women. When women are screened for the first time, prevalent disease will be detected. After undergoing at least one round of screening, the performance of different screening strategies may differ. The endline study was used to evaluate the CIN2+ incidence following a negative screen test to inform on frequency of screening in screen negative women. However, we understand this data is limited on the number of timepoints to measure CIN2+ incidence.

Note: We repeated analysis at endline to evaluate diagnostic accuracy of women who have already undergone a first round of screening as would happen in routine programmes (Supplementary Table 8). It was reassuring to verify that diagnostic accuracy was similar, although clearly the positive predictive value would be lower given the (relatively) low incidence of disease 16 months after a first screening

**2E).** It would be good for authors to refer to the TRIPOD statement to the principles of diagnostic and prognostic studies.

**Response**: We refer to the Moons et al, 2015 paper (*Ann Intern Med.* 2015;162:W1-W73), the TRIPOD statement “aims to improve the reporting of studies developing, validating, or updating a prediction model, whether for diagnostic or prognostic purposes”.

We wish to clarify that our study did not aim to develop or validate a diagnostic prediction model, but to evaluate existing diagnostic accuracy of screening and screening-triage approaches for detection of CIN2+ in different settings, and separately to evaluate risk of incident CIN2+ in screen-negative women.

We have reported according to the Standards for Reporting Diagnostic Accuracy (STARD) guidelines and have completed the STARD checklist, according to manuscript guidelines.

3) ROC curve is very important for the reporting of diagnostic tests. However, it's not mentioned at all in the statistics analysis section in methods. In Figure 2 and 3, there needs to be curves there not just the dots, and ROC values need to have 95% CIs. Also, the ROC values with 95% CI need to be presented in all the tables for test performance.

**Response:** The diagnostic tests evaluated are binary and therefore will appear as dots on the graphs and curves cannot be produced. The AUC is estimated from the single value using a standard method in Stata. On retrospect we feel the figures are sufficiently clear without calculating AUC values and therefore to avoid confusion we have now omitted the AUC values and not referred to figures 2 and 3 as ROC curves. We have added 95% Cis to figures 2 and 3 as requested.

4) In statistics analysis of the methods section, it says "We modelled the diagnostic accuracy of restricted genotyping approach using results of INNO-LiPA genotyping assay in the following combinations...", however, it's not clear what exactly the statistical model being used for this?

**Response:** We apologise for any confusion resulting from wording used. We used a combination of genotyping data (using INNO-LIPA genotyping assay) and Hybrid Capture II to simulate or replicate what could be achieved with a test similar to that of GeneXpert. We made the following change (line 152):

*We evaluated the diagnostic accuracy of restricted genotyping approach using results of INNO-LiPA genotyping assay in the following combinations …*

5) Table 1. P-value is missing for the variable "HIV-1 PVL in ART users".

**Response:** This has now been rectified in Table 1.

-----------------------------------------------------------

**Reviewer #2:** Thank you for this manuscript. This is a very important topic and a well written manuscript. In this time when the need for a specific cervical pre-cancer/cancer screening and prevention strategy for women living with HIV, such a research and manuscript adds to the accumulating evidence to inform policy making. I have very few comments and suggestions to help improve the manuscript:

1. line 9: I suggest you make "cervical screening" to read cervical cancer screening. This will be clearer to the readers and also consistent with the rest of the manuscript

**Response:** Thank you, we have revised the sentence on line 9.

2. Line 64-54: rephrase possible to read 'All participants were referred for colposcopy performed AT A median OF 12 weeks (interquartile range [IQR]: 8-15)...'

**Response:** Thank you, we have revised the sentence in line 78.

3. tables 2-5: I understand you have tried to put all the required information on the tables but as they look now, they are difficult to read. Kindly see what can be done to improve the legibility. I think they are very important tables and so any efforts to make them more legible is important.

**Response**: We agree with Reviewer 2 (and Reviewer 3 who makes a similar comment). We have simplified the Tables by combining data from both counties for HPV-DNA and VIA (country-level diagnostic accuracy data [previous Tables 2-5] have been added as Supplementary information, tables 1-4). For cytology however, given the differences in diagnostic accuracy (associated with differing level of experience and infrastructural to conduct cervical cytology in the two countries), we have presented the data separately by country throughout. Where country-level data differs between countries, this is summarised in the narrative results.

4. line 425: it should read '.....test positivity IS established.....'

**Response:** Thank you, we have revised the sentence in line 570.


-----------------------------------------------------------

**Reviewer #3:** The authors tackle a key unanswered question for women living with HIV (WLHIV) globally. Cervical cancer has become the leading cause of death for WLHIV with access to ART in Africa, but achievable strategies to mitigate this burden have been illusive due to challenges of scale, prevalence of dysplasia, and pathology/cytology/PCR capacity. In this project, they prospectively enroll WLHIV in Burkina Faso and South Africa (illustrative of the diversity of HIV/HPV prevalence and diagnostic capacities present where bulk of WLHIV live) to rigorously assess performance characteristics of visual, cytologic, and molecular approaches to screening. The core finding that molecular testing is superior (or equivalent to high performing cytology program) is a key piece of knowledge to move field/programs forward. However, as currently presented these findings are challenging for a general audience to gasps and understand importance. Revision for improved readability and focus could greatly improve impact.

Suggestions:

Major.
1) Improve readability of abstract/manuscript for general medical audience- Abstract reads as though two different studies (BF and RSA), but could be improved by noting that evaluating in low and high/middle-income contexts with differing HIV and HPV prevalences for improved generalizability. Suggest considering merging findings as seems like molecular testing optimal strategy in both settings (marginal difference with cytology in RSA), for detecting cancers and reducing specialist-dependant services.

**Response**: We agree with Reviewer 3 (as for Reviewer 2, comment 3). We now present diagnostic accuracy data combining the two countries in the main manuscript (abstract and starting from line 213). For HPV-DNA and VIA based approaches, the diagnostic accuracy is similar in both countries. However due to the large difference in diagnostic accuracy of cytology in both countries, we presented them separately using cut-off with best combination of sensitivity and specificity in each setting (ASCUS+ in Burkina Faso and HSIL+ in South Africa).

We retain the country stratified analyses in Supplementary information (supplementary tables 1-4) due to the different epidemiology of HPV infection and CIN2+, and differing approaches to cervical cancer screening (i.e. standard of care differs by country) in Burkina Faso and South Africa.

Similarly, due to similarity in diagnostic accuracy findings for the analysis of diagnostic accuracy stratified by both ART and age, we present country-combined data (beginning lines 291 and 333, respectively).

2) More directly assess impact of weaknesses on findings/confidence. The key weaknesses include old study (collected 2011/12, still reflective of current environment?), unblinding of pathologists (how much bias would need to be present to modify findings?), use of careHPV (challenging technology to use, and endpoints seem to only be HC-II done in Europe, reflective of reality in BF/RSA implementation?), does different geography of HPV prevalence affect the conclusions, and can any intervention be effective if takes 8-14mo to treat dysplasia and 37% got no treatment?

**Response**: We respond to the individual points below:

- Representativeness of study population and period effect

WLHIV included in this study were recruited from 2010 to 2012, at a time when they may have started ART according to older guidelines. As the CD4+ count at time of ART initiation, and history of immunosuppression, are important predictors of CIN2+CIN3+ and ICC incidence^[1]^, women included in this study may not be representative of contemporary or future cohorts of WLHIV in the universal ART era. However, our analysis of diagnostic accuracy according to ART status and duration attempted to correct for this period effect by restricting to women controlling HIV (prolonged ART and high CD4+) which corresponds to that recently observed in a contemporary cohort of WLHIV enrolled between 2013-2015 in the WIHS cohort in the USA^[2]^. We have added this limitation on line 550.

*Limitations of our study are that WLHIV included in the study may not reflect a contemporary cohort of WLHIV initiating ART in universal ART era and we did not evaluate HPV tests currently available in study settings today.*

Unblinding of pathologists:

**Response**: In our study, the histopathologists were blinded to the index test results (VIA, VILI, cytology and HC-II HPV test) but unblinded to colposcopy findings, as per routine local practice. However, since only women who had at least one abnormal result (any of VIA, VILI, cytology ASCUS+, Hybrid Capture-II positive) underwent biopsy, pathologists may have understood the high possibility to have abnormal findings.

We used a pragmatic approach, in that 4 quadrant biopsies (colposcopy directed and random biopsies of colposcopy normal quadrants) were taken to minimise verification bias. Our biopsy strategy led to 95% of women being biopsied in SA and 56% in BF. In the case of SA therefore the knowledge of biopsy could not orientate pathological diagnosis. In addition, local pathologist diagnoses were subjected to a strict review of endpoint by a consensus committee, which resulted in only a minority of case reclassification (this is referenced on line 548).

We also refer to line 107:

*The histo-pathologist was blind to VIA/VILI, cytology and HPV-DNA test results but was aware of colposcopy diagnosis.*

- Use of careHPV

We agree with Reviewers points and have added the following on line 556:

*We did* *not evaluate HPV tests currently available in local study settings and HC-II testing was conducted in France due to challenges in acquiring the CareHPV assay in time for study initiation. For reasons of harmonisation we decided to conduct all HPV testing at baseline in France due to lack of access to this technology in. CareHPV was conducted locally at study sites at endline after it had been acquired, staff were trained and Quality Control systems put in place. We conducted a formal evaluation of CareHPV compared to HC-II locally, which showed equivalent diagnostic accuracy as HC-II in a head-to-head comparison, previously published^[3]^.*

- Does different geography of HPV prevalence affect the conclusions:

**Response**: Given the high HPV prevalence in both countries, the sensitivity of HPV testing was higher and the specificity was low, and this was similar in both countries. However, specificity of HC-II was slightly lower in SA compared to BF. HPV prevalence was higher in South Africa compared to Burkina Faso and this was associated with HIV-related factors including lower likelihood of ART adherence, higher HIV-1 plasma viral load and lower CD4+ count. Women in South Africa also had higher prevalence of risk factors for HR-HPV and CIN2+ (hormonal contraception use, co-existence of other STI) compared to women in Burkina Faso, as previously reported (Kelly et al, AIDS 2017;31(2):273-285.). However, we stratified diagnostic accuracy by ART status (long duration, short duration and ART-naïve, corresponding with increasing HR-HPV and CIN2+ prevalence, line 333) and found that HC-II had higher specificity in prolonged ART users corresponding with HIV-1 viral suppression, higher CD4+ count and lower HR-HPV in these women. These findings were similar in both countries. Further, the predictive values and referral rates are influenced by positivity rates that differ in each locale.

- Can any intervention be effective if takes 8-14mo to treat dysplasia and 37% got no treatment

**Response**: We did not aim to measure the effectiveness of the intervention in this study, and for our research purposes, efforts were made to review women rapidly. It worked better in BF than SA due to general workload of services. Moreover, the need to have a biopsy confirmation incurred delays before treatment. In real practice, completion of screening and rapid management will depend on whether there is a need for a confirmatory test – this is a programmatic decision that could not be evaluated in this project, although the significant delays incurred in SA point to the need to have a more rapid and effective ‘test and treat’ strategy.

Our experience is useful in the context of SA and overwhelmed services where a quick test and treat approach might be more effective than anything that involves pathology services and frequent visit to overwhelmed gynaecology services.

3) Age triage. Many programs considering threshold of 30yrs to improve specificity for HPV testing. Does this subgroup have different performance characteristics>

**Response**: We assume the reviewer is referring to screening programmes in high income countries among women in the general population where the HPV prevalence is often much higher in younger women meaning the specificity of HPV testing would be low. The youngest women included in our study were aged 25 years and we observed higher HPV prevalence and consequently lower specificity of Hybrid Capture II in these women. We have added an age stratified analysis for each of the screening strategies, including triage options which is summarised on line 291 and Supplementary Table 5.

In summary, HC-II specificity was lowest in women in the youngest age group (25-29 years) and a triage strategy is most needed in these women if HPV based test is used in screening. Triage using a combination of HPV16/18 genotyping and HSIL+ (of non HPV16/18 positive women) in SA and HPV16/18 genotyping and VIA (of non HPV16/18 positive women) in BF resulted in the best combination of sensitivity and specificity for CIN3+ and reduced the number of colposcopy referrals.

Minor
1) Unsure why some patients got VIA or VILI or both

**Response**: We apologise for the confusion. All enrolled women had both VIA and VILI examinations. VIA was performed first, followed by VILI. We have re-phrased in Methods, line 75:

*All participants had a visual inspection with both acetic acid (VIA) and Lugol’s iodine (VILI) performed by trained nurses following the International Agency for Research on Cancer (IARC) guidelines.*

As all women had both VIA and VILI exam, we conducted an analysis on the diagnostic accuracy of VIA/VILI co-testing, whereby a woman was considered screen positive if she had a positive test result for either VIA or VILI and considered screen-negative if negative for both VIA and VILI. This is detailed in Methods, line 109. VIA on its own was considered as a strategy as some countries might only use this as a screening strategy.

2) For an HIV audience, they would expect viral suppression as a categorical variable rather than continuous. And proportion with viral failure on ART (often >1000 copies)

**Response**: We apologise for this omission. We have now provided the proportion of ART users with HIV-1 viral suppression (86.8% in Burkina Faso and 80.8% in South Africa), and separately median HIV-1 viral load among ART-naïve in Table 1.

3) Introduction and elsewhere suggest relationship between HPV vaccination and HPVtesting, but not related

**Response**: Currently, adult WLHIV are not targeted in HPV vaccination programmes in either country, although they could benefit from HPV vaccination, and screening is the only available intervention available for cervical cancer prevention to these women. HPV testing could be a triage strategy to vaccinate women even if older than traditional ages for vaccination, similar to what has been observed in older women in the general population (HPV-FASTER).

4) Discussion long and lots of extrapolations re: CD4, ART timing beyond the data of this project. **Response**: We have made revisions to shorten the Discussion.

-----------------------------------------------------------

Additional changes not directly related to Reviewer comments:

We have added two additional strategies for triage of HPV positive women which are of interest in the field: HPV-> HPV16/18 & HSIL+ (i.e. HPV primary test with reflex HPV16/18 genotyping and cytology [HSIL+] of non-HPV16/18 women) and HPV -> HPV16/18 & VIA (i.e. HPV primary test with reflex HPV16/18 genotyping and VIA of non-HPV16/18 women) as these test strategies are being increasingly considered for triage of HPV positive women in the general population, and among WLHIV.

We have added the following to methods (line 138):

*Among HR-HPV (HC-II) positive women, we evaluated the diagnostic accuracy of triage approaches, including: VIA, VIA/VILI, cytology (ASCUS+ and HSIL+), a combination of HPV16/18 genotyping and cytology (test positive if HPV16 or HPV18 positive, or cytology [HSIL+] when negative for both HPV16 and HPV18) and combination of HPV16/18 genotyping and VIA (test positive if HPV16 or HPV18 positive, or VIA abnormal when negative for both HPV16 and HPV18).*

**Requests from the editors (writeup/formatting changes for the journal):**

*Please structure your abstract using the PLOS Medicine headings (Background, Methods and Findings, Conclusions) - "methods and findings" is a single subsection. In the last sentence of the Abstract Methods and Findings section, please include a brief note about any key limitation(s) of the study's methodology.
**Response: This has now been updated, and limitations been added (as below).**

*Limitations of our study are that WLHIV included in the study may not reflect a cotemporary cohort of WLHIV initiating ART in universal ART era and we did not evaluate HPV tests currently available in study settings today.*

*It would also be good to include in the Abstract section some information about the dates of enrollment into the two cohorts involved in this study. If length is an issue for the abstract (with more methods info and limitations now mentioned), some of the less informative results could be omitted.
**Response:** We have add the following in the Abstract “WLHIV aged 25-50 attending routine HIV treatment centres in Burkina Faso (BF) and South Africa (SA) from December 2011 to October 2012 were enrolled in a prospective evaluation study”

*At this stage, we ask that you include a short, non-technical Author Summary of your research to make findings accessible to a wide audience that includes both scientists and non-scientists. The Author Summary should immediately follow the Abstract in your revised manuscript. This text is subject to editorial change and should be distinct from the scientific abstract. Please see our author guidelines for more information: [https://journals.plos.org/plosmedicine/s/revising-your-manuscript#loc-author-summary](https://protect-eu.mimecast.com/s/VhI8CqZ14tOL4QYf2nTMv?domain=journals.plos.org)

**Response: This has now been added.**

*It would be good to clarify in the paper whether the analytical approach reported here corresponded to one laid out in a prospective protocol or analysis plan. Please state this (either way) early in the Methods section.

a) If a prospective analysis plan (from your funding proposal, IRB or other ethics committee submission, study protocol, or other planning document written before analyzing the data) was used in designing the study, please include the relevant prospectively written document with your revised manuscript as **a Supporting Information file** to be published alongside your study, and cite it in the Methods section. A legend for this file should be included at the end of your manuscript.

b) If no such document exists, please make sure that the Methods section transparently describes when analyses were planned, and when/why any data-driven changes to analyses took place.
c) In either case, changes in the analysis-- including those made in response to peer review comments-- should be identified as such in the Methods section of the paper, with rationale.

**Response**: We have uploaded the statistical analysis plan for the HARP study analysis (reported to funder, the European Commission). The analysis reported in this manuscript corresponds to HARP Study 1, Objective 1 : to determine the relative diagnostic performance of a novel high-risk (HR) HPV DNA rapid test (*CareHPV*) versus other screening tests (cytology, VIA/VILI) to detect high-grade cervical intraepithelial neoplasia (CIN2+) , among HIV-infected African women attending HIV care settings. A secondary analysis was to evaluate the diagnostic performance for CIN3+ detection.

*The paper is clear that the STARD guideline was used in reporting (and the checklist is appended as a supporting information file) - however we'd recommend that the authors call out the SI file corresponding to the checklist in the Methods section where this is mentioned. In addition, some of the STARD items are blank, and some of these correspond to issues noted by one reviewer as being unclear in the study, so it would be good to clarify this. Finally when completing the checklist for resubmission, please use section and paragraph numbers, rather than page numbers (because the latter are amended when the paper is reformatted for publication).

**Response**:

- We have made reference to the STARD checklist in Methods, line 172:

*This Article was reported according to the Standards for Reporting Diagnostic Accuracy (STARD) statement (Supplementary Figure 1*)

- We refer to the STARD checklist in the list of supporting files at the end of the manuscript (final page).
- The STARD checklist has been updated to include section heading and paragraph number, and brief description responding to each item.
  -----------------------------------------------------------

References

1.	Kelly H, Weiss HA, Benavente Y, de Sanjose S, Mayaud P, Qiao Y-l, et al. Association of antiretroviral therapy with high-risk human papillomavirus, cervical intraepithelial neoplasia, and invasive cervical cancer in women living with HIV: a systematic review and meta-analysis. The Lancet HIV. 2018;5(1):e45-e58.

2.	Strickler HD, Keller MJ, Hessol NA, Eltoum I-E, Einstein MH, Castle PE, et al. Primary HPV and Molecular Cervical Cancer Screening in US Women Living with HIV. Clinical Infectious Diseases. 2020.

3.	Ngou J, Magooa MP, Gilham C, Djigma F, Didelot M-N, Kelly H, et al. Comparison of <em>care</em>HPV and Hybrid Capture 2 Assays for Detection of High-Risk Human Papillomavirus DNA in Cervical Samples from HIV-1-Infected African Women. Journal of Clinical Microbiology. 2013;51(12):4240-2.
