# Supplementary material for: Diagnostic accuracy of cervical cancer screening and screening–triage strategies among women living with HIV-1 in Burkina Faso and South Africa: A cohort study
Source: PLoS Med. 2021 Mar 4;18(3):e1003528. doi: 10.1371/journal.pmed.1003528 (PMC7971880; doi:10.1371/journal.pmed.1003528)
Supplement: Attachment — Submitted filename: Letter to editor_161220.docx [file pmed.1003528.s014.docx]

**Re: PMEDICINE-D-20-04079R2: Diagnostic accuracy of cervical cancer screening and screening-triage strategies among women living with HIV-1 in Burkina Faso and South Africa: A cohort study**

16 December 2020

To the Editor of PLOS Medicine,

We wish to thank the Editors and Reviewers for the acceptance of the manuscript “**Diagnostic accuracy of cervical cancer screening and screening-triage strategies among women living with HIV-1 in Burkina Faso and South Africa: A cohort study”** (reference number: **PMEDICINE-D-20-04079R2**).

We are re-submitting a revision of the manuscript with the requested Editorial changes, as detailed in the remainder of this letter. [Our responses are highlighted in blue.]

Please note that when we discuss revisions made to the manuscript, the location of the revision is indicated by line number in the non-track mode version of the manuscript.

Thank you for your consideration of this revised manuscript,

Yours sincerely,

Helen Kelly, on behalf of all co-authors

------------------------------------------------------------
Requests from Editors:

1. Title: Please revise to: “Diagnostic accuracy of cervical cancer screening and screening-triage strategies among women living with HIV-1 in Burkina Faso and South Africa: A cohort study”

**Response:** This has now been revised.

2. Please provide the data underlying your study, and update the Data Availability Statement accordingly. We will need to check this prior to acceptance.

**Response:** The de-identified patient level data has been uploaded on Editorial Manager in Excel format (filename: HARP_DTA_PMED_111220) and also publicly available at on Mendeley online repository at <http://dx.doi.org/10.17632/yd5ygw38vj.1> . The data is under embargo until 01 January 2021 and has CC BY 4.0 licence.

The following has been added on line 236:

*“The dataset is available at Mendeley online repository at doi:10.17632/yd5ygw38vj.1. “*

3. Please include line numbers in the margin throughout, including the abstract and author summary.

**Response:** This has now been revised.

4. Throughout, please use the following capitalisation: "sub-Saharan”.

**Response:** This has now been revised.

5. Abstract:
a. Please give more summary demographic information, eg. median age at recruitment, prior screenings.

**Response:** We have added (line 12):
*Among 1238 women (BF:615; SA:623), median age was 34 and 36 years in BF and SA (p<0.0001), 28.6%/49.6% ever had prior cervical cancer screening (p<0.001) and 69.9%/64.0% were taking ART at enrolment (p=0.045).*

We have also added an additional row in Table 1 for “Ever had prior cervical cancer screening, n (%)”

b. Please include p values for comparisons.

**Response:** Added, as detailed above in line 12.

c. Please include 95% CIs for these results: “CIN2+ incidence over 16 months was highest among VIA baseline screen-negative women (2.2%) and HC-II triage with VIA (2.1%) and lowest among HC-II screen-negative women (0.5%).”

**Response:** This has now been revised.

d. We had some difficulty locating the data in these statements for verification; please indicate which Table these can be found in:
**Response:** We apologise for the confusion. The data refers to country specific findings which were moved to Supplementary information as part of the previous revisions. We have referenced the data source in **bold blue** below. We have also made some small changes for clarity, as indicated (underlines):

i. “Triage of HC-II positive women with VIA/VILI reduced the number of colposcopy referrals, but with loss in sensitivity for CIN2+ (58.1%; **Supplementary Table 3**) but not for CIN3+: (84.6%; **Supplementary Table 1; and line 316**).”

ii. “HC-II had higher sensitivity (86.8%, 95%CI: 79.7-92.1) but low specificity (48.2%, 95%CI; 43.5-53.0) resulting in almost twice as many referrals.”

We apologise for the slight error. The data in the abstract refers to HC-II sensitivity and specificity for CIN2+ detection in SA (which is provided in Supplementary Table 4 ) while in the main text of the manuscript (line 305), we refer to HC-II sensitivity and specificity for CIN3+ detection in SA. For consistency, we have changed the data in the abstract to match what is reported in line 305 :

*“HC-II had similar sensitivity for CIN3+ (83.0%, 95%CI: 70.2-91.9) but low specificity (42.7%, 95%CI: 38.4-47.1) resulting in almost twice as many referrals.”*

iii. “Compared to HC-II, triage of HC-II positive women with HSIL+ resulted in 40% reduction in colposcopy referrals but was associated with some loss in sensitivity.”

This sentence refer to data in line 319 (and Supplementary Table 2). We have added the following (underlined) for further clarification in line 319:

*In SA, triage of HC-II positive women with cytology HSIL+ increased the number of referrals compared to HSIL+ alone from 301 to 363 per 1000 women but referral rate was 40% lower than using HC-II alone (597 per 1000 women) and while sensitivity for CIN3+ was high (88.4%, 95%CI: 74.9-96.1;* ***Supplementary Table 2****), this approach would miss 26.9% (14/52) of women with CIN3+ due to lower sensitivity of HC-II as a screen test.*

e. Conclusions: Please start with “In this cohort study, …”
f. Conclusions: Please mention WLHIV as well.
**Response:** We have re-written the abstract conclusion as follows:

***Conclusions****: In this cohort study among WLHIV in Africa, an HPV test targeting 14 HR types had higher sensitivity to detect CIN2+ compared to visual inspection but had low specificity, although a restricted genotype approach targeting 8 HR types decreased the number of unnecessary colposcopy referrals. Cytology HSIL+ had optimal performance for CIN2+/CIN3+ detection in SA. Triage of HPV positive women with HSIL+ maintained high specificity but with some loss in sensitivity compared to HC-II alone.*

6. Author summary:
a. Please add “(WLHIV)” after first use.

**Response:** This has now been revised.

b. Please correct: “precancerous lesions cervical” to “precancerous cervical lesions”

**Response:** This has now been revised.

c. Please define/clarify “cytology” and “colposcopy” to a lay reader.

**Response:** We have added the following to line 52:

“Screening approaches most commonly used in sub-Saharan Africa using visual inspection of the cervix or cervical cytology which checks for cervical cell abnormalities have shown variable diagnostic accuracy to detect precancerous lesions. Molecular-based screening approaches, including HPV-DNA strategies which screen for oncogenic HPV infection have shown high sensitivity for cervical precancer but can result in over referral to colposcopy, a procedure to determine eligibility for treatment. “

d. Please avoid the phrasing “false-positive”, and instead merely describe this subset of patients, eg …infections that have not progressed to a cytology-detectable lesion…“”. Please also clarify the “second test” as being also for HPV.

**Response:** We have re-phrased the following in line 68:

“We found that an HPV-DNA based test identified a greater number of women with precancer compared to visual inspection and cytology methods. However, there was a greater proportion of women without precancer who had a positive HPV-DNA test result, meaning a triage test using an alternative method (i.e. cytology or visual inspection) was required to determine treatment eligibility. “

e. Please define “ART” at first use.

**Response:** This has now been revised.

f. Please delete “and in COVID-19 era”
**Response:** This has now been revised.

7. Please make your reference citations not superscript, and include a space between the text and the brackets, eg: “…increase screening coverage and uptake [1].” Please remove spaces from within citations themselves.

**Response:** This has now been revised. Although it is not clear the meaning of “ Please remove spaces from within citations themselves “. We can rectify once clarified.

8. Line 54: Please include days in the recruitment dates.

**Response:** This has now been added (line 145).

9. Line 90: Please remove registered trademark symbol.

**Response:** This has now been deleted (line 181).


10. PLOS does not permit "data not shown” (eg. lines 176, 264, 293). Please remove this claim, or do one of the following:
a) If you are the owner of the data relevant to this claim, please provide the data in accordance with the PLOS data policy, and update your Data Availability Statement as needed.
b) If the data not shown refer to a study from another group that has not been published, please cite personal communication in your manuscript text (it should not be included in the reference section). Please provide the name of the individual, the affiliation, and date of communication. The individual must provide PLOS Medicine written permission to be named for this purpose.
c) For any other circumstance, please contact the journal office ASAP.

**Response:** We have now provided the individual patient data in an excel spreadsheet (filename HARP_DTA_PMED_111220). Although the analyses with reference “data not shown” (lines 267, 367, 394) refer to analyses conducted but not tabulated in the main manuscript or supplementary tables, it is possible to replicate the analysis with the raw data in the excel files. If this is still not sufficient, we can reproduce the analyses in table form and add to supplementary tables. We have deleted ‘data not shown’ in lines 267, 367, 394.


11. Table 1 and throughout: Please show exact p values over 0.001, or p<0.001 otherwise.

**Response:** We have revised.

12. Line 346: Typo “..”

**Response:** This has now been corrected (line 448).


13. Page 27: Please remove the declaration of interests and author contributions – these are taken from the online submission form.

**Response:** This has now been deleted.

14. Please double check and correct the URLs for references 1, 2, 18.

**Response:** We apologise for these errors. References 1, 2 have and 18 have been changed to correctly link to appropriate reference.

15. Please provide a URL or DOI for references 11 and 33.

**Response:** This has now been rectified. For reference 33, the full citation is now added but in case it is required, the doi is <https://doi.org/10.3389/fonc.2014.00048>


16. Please remove duplicate URLs from references 14 and 15.

**Response:** Reference 14 has been updated to more recent data, reference 15 has been corrected.

17. Please include a title for reference 22.

**Response**- This is the full citation, as per PubMed : SMITH, J.H.F.. (2002), Bethesda 2001. Cytopathology, 13: 4-10. <https://doi.org/10.1046/j.1365-2303.2002.00397.x>

This is as currently cited in the manuscript.

18. Please list the short journal name for “PLoS ONE” consistently in references.

**Response**- This is now corrected.

19. Please rename the STARD checklist file as “S1 Checklist”. Line 153: Please update the reference to this file.

**Response**- This is now corrected (line 244)


20. Please rename the analysis plan file as “S1 Text”. Please cite this file in the Methods, as with the STARD checklist.

**Response**- Filename has been corrected. Reference to the analysis plan is now added (line 243) and also mentioned in S1 Checklist (Item 12).

---

Additional changes:

- In the no-track mode of the previous revision, the text for the paragraph “Diagnostic accuracy of screening strategies by age at screening” (Line 342-351) was inadvertently deleted, however it had been retained in the ‘track’ version submitted. In the current R2 version, it has been re-inserted.
- Line 360: The role of HIV related factors on diagnostic accuracy of screening strategies. We have used data on specificity for <CIN2 (rather than specificity for <CIN3) as we consider this more clinically relevant. Supplementary Table 7 has also been updated to include data for outcome of CIN2+ as well as CIN3+.

End of comments

**********************
